# Role of microRNAs and Exosomes in *Helicobacter pylori* and Epstein-Barr Virus Associated Gastric Cancers

**DOI:** 10.3389/fmicb.2018.00636

**Published:** 2018-04-05

**Authors:** Iva Polakovicova, Sofia Jerez, Ignacio A. Wichmann, Alejandra Sandoval-Bórquez, Nicolás Carrasco-Véliz, Alejandro H. Corvalán

**Affiliations:** ^1^Advanced Center for Chronic Diseases, Pontificia Universidad Católica de Chile, Santiago, Chile; ^2^UC Center for Investigational Oncology, Pontificia Universidad Católica de Chile, Santiago, Chile; ^3^Department of Hematology-Oncology, Faculty of Medicine, Pontificia Universidad Católica de Chile, Santiago, Chile

**Keywords:** gastric cancer, Helicobacter pylori, Epstein-Barr virus, microRNA, Exosomes, lncRNA (long non-coding RNA)

## Abstract

Emerging evidence suggests that chronic inflammation caused by pathogen infection is connected to the development of various types of cancer. It is estimated that up to 20% of all cancer deaths is linked to infections and inflammation. In gastric cancer, such triggers can be infection of the gastric epithelium by either *Helicobacter pylori* (*H. pylori*), a bacterium present in half of the world population; or by Epstein-Barr virus (EBV), a double-stranded DNA virus which has recently been associated with gastric cancer. Both agents can establish lifelong inflammation by evolving to escape immune surveillance and, under certain conditions, contribute to the development of gastric cancer. Non-coding RNAs, mainly microRNAs (miRNAs), influence the host innate and adaptive immune responses, though long non-coding RNAs and viral miRNAs also alter these processes. Reports suggest that chronic infection results in altered expression of host miRNAs. In turn, dysregulated miRNAs modulate the host inflammatory immune response, favoring bacterial survival and persistence within the gastric mucosa. Given the established roles of miRNAs in tumorigenesis and innate immunity, they may serve as an important link between *H. pylori*- and EBV-associated inflammation and carcinogenesis. Example of this is up-regulation of miR-155 in *H. pylori* and EBV infection. The tumor environment contains a variety of cells that need to communicate with each other. Extracellular vesicles, especially exosomes, allow these cells to deliver certain type of information to other cells promoting cancer growth and metastasis. Exosomes have been shown to deliver not only various types of genetic information, mainly miRNAs, but also cytotoxin-associated gene A (CagA), a major *H. pylori* virulence factor. In addition, a growing body of evidence demonstrates that exosomes contain genetic material of viruses and viral miRNAs and proteins such as EBV latent membrane protein 1 (LMP1) which are delivered into recipient cells. In this review, we focus on the dysregulated *H. pylori*- and EBV-associated miRNAs while trying to unveil possible causal mechanisms. Moreover, we discuss the role of exosomes as vehicles for miRNA delivery in *H. pylori*- and EBV-related carcinogenesis.

## Gastric cancer as a worldwide problem

Gastric cancer (GC) is one of the most common cancers worldwide (Ferlay et al., 2015). GC occupies the fifth place in incidence, with more than 900,000 new cases every year, representing roughly 6.8% of the global cancer cases. GC is one of the deadliest types of tumors. In 2012, it ranked third as cause of cancer-related death, with 723,000 deaths. The highest estimated mortality rates are found in Eastern Asia and Latin America, and the lowest in North America and Australia (Torre et al., 2015). More economically developed countries, like Japan, have low mortality rates due to better screening programs (Ajani et al., 2017). In recent years, Latin America has also shown a decreasing trend in mortality, and it is expected that global GC mortality will decline in the future (Chatenoud et al., 2014).

**Figure 1:**
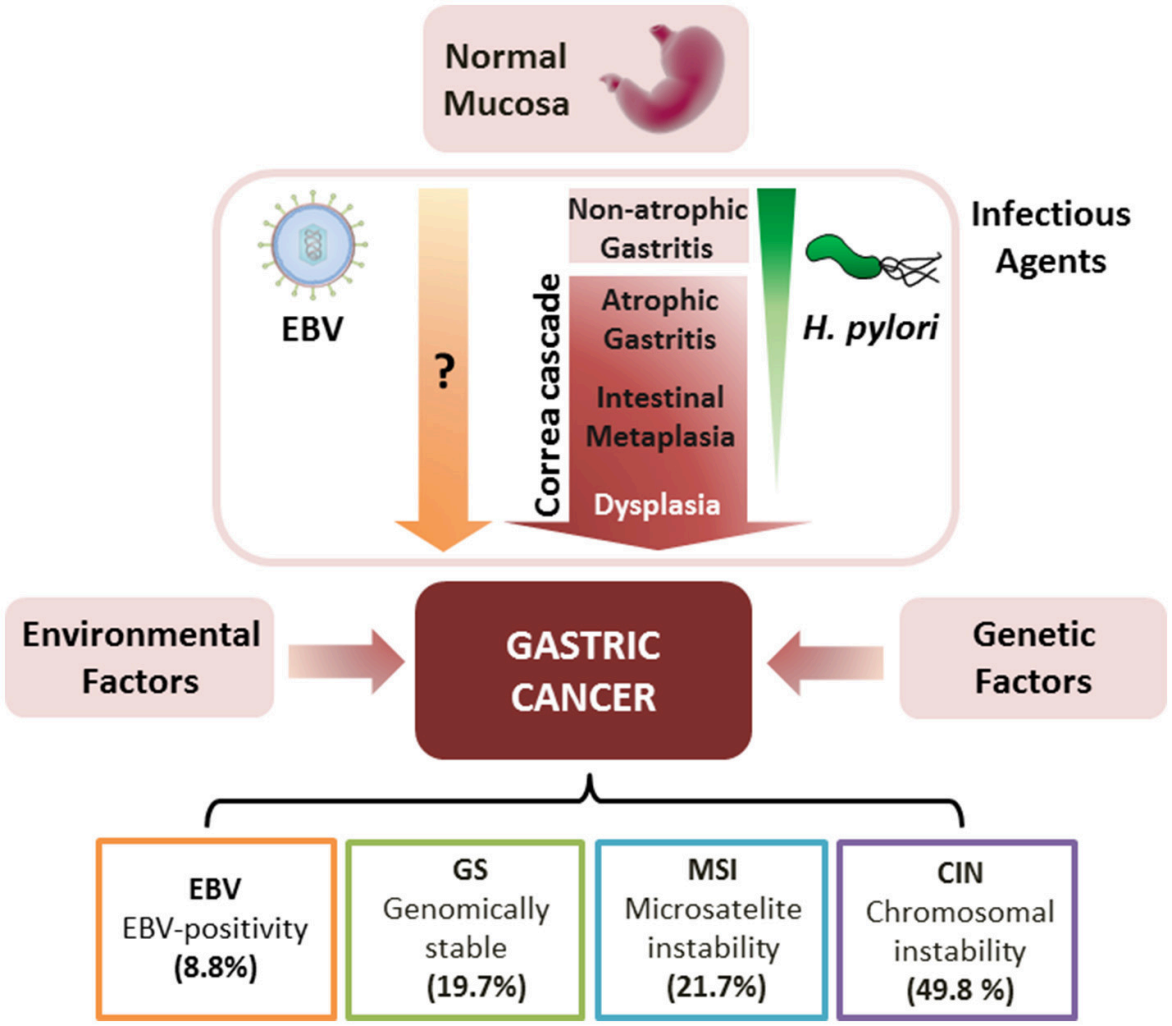
Gastric carcinogenesis, in context of chronic infection (*H. pylori* and EBV), and TCGA classification of gastric cancer. *H. pylori* colonizes normal mucosa inducing non-atrophic gastritis and the precancerous “Correa Cascade,” consisting of multifocal atrophic gastritis, intestinal metaplasia and dysplasia. *H. pylori* is usually lost during the progression of these lesions (fading green triangle). This process can be complemented by host-dependent genetic and environmental factors leading to gastric cancer. Alternatively, EBV can lead to the development of gastric cancer in an unknown manner. According to TCGA, gastric cancer tumors can be classified by (i) EBV-positivity (EBV), (ii) genomically stable (GC), (iii) microsatellite instability (MSI), and (iv) chromosomal instability (CIN). Percentage of each subtype is shown.

Several morphological and novel molecular classifications have been developed for the comprehensive characterization of GC (Hamilton and Aaltonen, 2000; The Cancer Genome Atlas Research Network, 2014). Among them, the histomorphological approach by Lauren (1965) recognizes two main types of gastric carcinomas: intestinal and diffuse. In the case of the intestinal-type GC, a long-standing preneoplastic process known as the “Correa cascade” begins with multifocal atrophic gastritis, progressing to intestinal metaplasia and cancer over the next 30–50 years (Carrasco and Corvalan, 2013; Sandoval-Bórquez et al., 2015). Infectious agents such as *Helicobacter pylori* (*H. pylori*) and Epstein-Barr virus (EBV), as well as host-dependent genetic [polymorphisms in interleukin (IL)-1β, TNF-γ, and IL-10] and environmental factors (i.e., smoking and alcohol consumption), play roles in the heterogeneity of this preneoplastic cascade (Figure 1). This review focuses on the role of (1) infectious agents, (2) non-coding RNAs (ncRNAs), mainly microRNAs (miRNAs) and long ncRNAs (lncRNAs), and extracellular vesicles, particularly (3) exosomes and (4) vesicles of bacterial origin; in the gastric precancerous cascade.

## Infectious agents in gastric cancer

### *H. pylori* infection

*H. pylori* is a Gram-negative spiral-shaped bacterium, present in the gastric epithelium of over 50% of the world's population (Jones et al., 2010). *H. pylori* infection has been associated with a variety of diseases including chronic gastritis, peptic ulcers, and epithelial and lymphoid malignancies of the stomach (Moss, 2017). Chronic infection by *H. pylori* is the strongest known risk factor for intestinal and diffuse histomorphological type GC (Helicobacter and Cancer Collaborative Group, 2001; Moss, 2017). *H. pylori* genome diversity and the presence of bacterial virulence factors play an important role determining the outcome of *H. pylori* infection. The cytotoxin-associated gene A (*cagA*) encodes CagA, the major virulence factor of *H. pylori*, which is delivered into gastric epithelial cells via bacterial type IV secretion. CagA positive and vacuolating cytotoxin A (VacA) with allelic variant s1m1 strains are associated with increased disease severity (Wroblewski and Peek, 2013).

Infection of the gastric mucosa by this pathogen causes inflammation of the gastric tissue (non-atrophic gastritis), which initiates the “Correa cascade.” The actual pre-neoplastic cascade begins at multifocal atrophic gastritis, characterized by loss of the gastric glands due to chronic inflammation (Correa, 1988). It is known that *H. pylori* inhabits the glandular epithelium of the stomach, and the bacterium is commonly lost during the progression of precancerous lesions as a result of the replacement of these glands for intestinal-like epithelium (intestinal metaplasia) (Morson, 1955). Therefore, the oncogenic role of *H. pylori* resides in the initial steps of the gastric precancerous cascade. Hence, this review summarizes studies on host ncRNAs (lncRNAs/miRNAs) which are dysregulated in association with *H. pylori* infection.

Eradication of *H. pylori* reduces overall GC rates, but only in early pre-cancerous lesions (i.e., non-atrophic and multifocal atrophic gastritis) and not in advanced lesions (i.e., intestinal metaplasia and dysplasia; Chen et al., 2016). Thus, whether to implement programs aiming for *H. pylori* eradication remains an open question.

### EBV infection

EBV is a linear, double-stranded DNA virus and a member of *herpesviridae* family. EBV was described more than 50 years ago in patients with Burkitt's lymphoma and was the first virus linked to cancer in humans (Young et al., 2016). EBV has a high prevalence worldwide and it is thought that in about 90% of adults, EBV establishes persistent infection (Cohen, 2000). EBV is best known as the cause of infectious mononucleosis in adolescence or young adulthood. EBV is linked to a variety of human tumors, including lymphoid (Burkitt's lymphoma, Hodgkin's disease, B cell lymphomas) and epithelial neoplasms [nasopharyngeal carcinoma (NPC) and GC; Young et al., 2016]. In the case of GC, EBV-associated gastric carcinoma (EBVaGC) is linked to the diffuse histomorphological type GC (Camargo et al., 2011; Carrasco-Avino et al., 2017). In addition, the effect of EBV infection on GC prognosis, evaluated in 4,599 patients by an international pooled analysis, shows that patients with EBV-positive tumors have increased overall survival rates than EBV-negative cases (Camargo et al., 2014).

Although the association between EBV and gastric carcinoma was first proposed in 1992 by Shibata and Weiss (1992), it took more than 20 years to be recognized (The Cancer Genome Atlas Research Network, 2014). The Cancer Genome Atlas (TCGA) consortium, in its novel molecular classification for GC, recognizes EBVaGC as one of the four proposed subtypes, representing about 9% of all gastric carcinomas (Figure 1). This subtype of GC harbors recurrent mutations in the PIK3CA gene, DNA hypermethylation, and amplifications of JAK2, CD274, PDCD1LG2, and ERBB2 (The Cancer Genome Atlas Research Network, 2014). Such characteristics imply altered proliferation, apoptosis and immune suppression and evasion (Sun et al., 2007). Interestingly, EBVaGC has been reported to possess the most extensive CpG island methylation on both human and viral genomes, which is more extensive than in any other tumor type in the TCGA database (Zouridis et al., 2012; The Cancer Genome Atlas Research Network, 2014; Gulley, 2015; Alarcon et al., 2017).

EBV infects B cells by recognition of CD21 on the cell surface, from where it is transported to the cell nucleus. After infection, EBV can enter into a lytic cycle or switch to a latency stage. The coding and non-coding latency genes repress the lytic cycle and specific sets of these genes are associated with malignancies. The expression patterns of coding and non-coding latency genes that repress the lytic cycle in EBV-associated malignancies is well characterized, except for EBVaGC. The most commonly observed pattern in this disease is latency II-like (44.4%), defined by expression of EBNA1, EBERs, BARF1, and LMP2A genes (zur Hausen et al., 2000). The second most common pattern is latency I (42.9%), which is restricted to EBNA1, EBERs, and BARTs. Latencies II and III represent only few cases (Price and Luftig, 2015; Ribeiro et al., 2017). Specific sections within this review explain the role of each of the above-mentioned genes. Clearly, further research is warranted to clarify the specific expression patterns of coding and non-coding latency genes in EBVaGC.

### *H. pylori* and EBV co-infection

Although *H. pylori* and EBV have been clearly identified as etiological agents of GC, only few studies have analyzed the role of co-infection in this process. The mechanism of interaction between *H. pylori* and EBV is not known. As both pathogens induce severe inflammatory response, chronic inflammation is a common theme. This observation is supported by a study in which co-infected patients presented enhanced inflammatory lesions compared to those only infected with *H. pylori* or EBV (Cardenas-Mondragon et al., 2013). Moreover, EBV positivity in *H. pylori* positive GC was associated to premalignant lesions and intestinal-type GC (Cárdenas-Mondragón et al., 2015). Thus, EBV probably collaborates with *H. pylori* through the induction of additive inflammatory response and enhanced inflammation.

Although only *H.pylori* is associated with the activation of E-cadherin/β-catenin/TCF-4 signaling pathway in normal gastric epithelial cells (Yu et al., 2011, 2014), both pathogens are linked to the activation of NF-κβ and MAP kinases oncogenic pathways in GC cell lines (Mohr et al., 2014; Liu and Cohen, 2015; Byun et al., 2016; Dela Pena-Ponce et al., 2017). Co-infection by EBV can also hinder the host response to *H. pylori*. Host protein SHP 1 interacts with *H. pylori* virulence factor CagA, causing dephosphorylation and inactivation of the bacterial protein. Thus, SHP 1 prevents oncogenic activity of CagA. However, EBV coinfection causes methylation of host SHP 1, silencing its expression. Consequently, the cooperative effect of EBV may increase the oncogenic potential of *H. pylori* CagA (Saju et al., 2016).

Epigenetic modifiers (DNMT1, DNMT3B, EZH2), tumor suppressor genes (RPRM, MGMT, TWIST1), miRNAs (let-7a/c, miR-155) and multiple signaling pathways are dysregulated in the process of gastric carcinogenesis (Schneider et al., 2010; Hayashi et al., 2013; Zhang et al., 2016). In intestinal-type GC, down-regulation of CDH1 by DNA methylation has been exclusively linked to the presence of CagA-positive *H. pylori* strains (Ferrasi et al., 2010). On the other hand, in EBV-positive GC, a distinct gene silencing pattern has been identified including p16, p14, APC, p73, RUNX3, MINT2, MINT31, FHIT, CRBP1, WWOX, and DLC-1, in spite of the extensive DNA methylation observed in these tumors (Geddert et al., 2011; Saito et al., 2013; He et al., 2015). CagA-positive *H. pylori* strains attenuate let-7a/c expression by histone modification and DNA methylation and lead to activation of the Ras pathway (Hayashi et al., 2013). Conversely, the EBV latent infection protein EBV nuclear antigen 1 (EBNA1) upregulates multiple let-7 family miRNAs, including let-7a in NPC, as well as GC cell lines. Cellular targets of let-7a, such as Dicer, are downregulated in both types of cell lines (Mansouri et al., 2014). *H. pylori* upregulates miR-155 (Xiao et al., 2009), which is a strong inhibitor of inflammatory response in EBV-associated tumors including lymphoid and epithelial cancers (Jiang et al., 2006; Du et al., 2011). Clearly, further studies are warranted to elucidate mechanisms driving commonalities in co-infection by both etiological agents of GC.

## ncRNAs and miRNAs

The non-coding genes make up roughly 98% of the human genome, and are involved in the regulation of multiple cellular processes. ncRNAs are defined as RNAs that do not code for proteins and are divided into small (~20–200 nucleotides) and long (lncRNAs) RNAs (200 nucleotides to ~100 kilobases; kb; Sana et al., 2012). Small ncRNAs include miRNAs, PIWI-interacting (piRNAs), ribosomal (rRNAs), transfer (tRNAs), small nuclear (snRNAs), and nucleolar (snoRNAs) RNAs (Hirose et al., 2014). Among small ncRNAs, miRNAs are highly conserved and regulate gene expression in multicellular organisms at a posttranscriptional level (Ambros, 2004). In GC, some ncRNAs can function as oncogenes or tumor suppressor genes by controlling the expression of target genes (Zhang et al., 2014, 2018).

### Host lncRNAs associated with *H. pylori* infection

lncRNAs make up the largest portion of the mammalian non-coding transcriptome (Derrien et al., 2012). lncRNAs regulate a plethora of cellular processes and aberrant expression of lncRNAs could play an important role in gastric carcinogenesis (reviewed in Li et al., 2016; Yoshida and Kimura, 2017). A few studies have explored the role of lncRNAs in GES-1, a normal epithelial gastric cell line, infected with *H. pylori*. Twenty-four hours post-infection, 23 and 21 lncRNAs were upregulated and downregulated, respectively. Specifically, lncRNAs XLOC_004562, XLOC_005912, and XLOC_000620 were highly expressed, whereas XLOC_004122 and XLOC_014388 were downregulated (Table 1). These latter two lncRNAs were validated in clinical samples positive for *H. pylori* infection and further re-expressed post *H. pylori* eradication (Yang et al., 2015). Profiling of multiple lncRNAs and coding transcripts in *H. pylori*-infected GES-1 cells has identified a network of multiple dysregulated lncRNA-mRNAs involved in the development of *H. pylori*-related pathologies, including GC (Zhu et al., 2015). Later studies have identified specific examples of this, such as decreased expression of lncRNA AF147447 in *H. pylori* infection. This non-coding transcript inhibits cell proliferation and invasion through downregulation of oncogene MUC2 (Zhou et al., 2016). More recent studies have begun to shed light on the transcriptional crosstalk between lncRNAs and mRNAs in GC, proving that this is a promising field in biomedicine (Zhang et al., 2018) which will broaden our understanding of the pathogenesis of this disease. Furthermore, tissue-specificity and ability to predict clinical subtypes of cancer by their future behavior suggest great potential for these ncRNAs as biomarkers for diagnosis and prognosis (Du et al., 2013; Schmitt and Chang, 2016).

**Table 1:** Deregulated host miRNAs and lncRNAs in *H. pylori* infection.

**Gene name**	**Type of noncoding gene**	**Variation**	**Associated deregulated genes**	**Type of specimen**	**Associated biological process**	**References**
let-7b	miRNA	down	IL-1β, IL-8	Clinical samples	Immune response	Isomoto et al., 2012
miR-21	miRNA	up	RECK	Clinical samples	Cell proliferation, invasion, migration and apoptosis	Zhang et al., 2008
miR-103	miRNA	down	IL-1β, IL-6, IL-8 and TNF-α	Clinical samples	Immune response	Isomoto et al., 2012
miR-124	miRNA	down	SMOX	Cell lines	DNA damage and tumorigenesis	Murray-Stewart et al., 2016
miR-155	miRNA	up	Tspan14, Lpin1, Pmaip1 and PKIα	Clinical samples clinical	Immune response and apoptosis	Fassi Fehri et al., 2010; Koch et al., 2012
			IKK-ε, SMAD2, FADD	Samples and cell lines	Proinflammatory cytokines	Xiao et al., 2009
miR-141	miRNA	down	IL-1β, IL-6, IL-8 and TNF-α	Clinical samples	Immune response	Isomoto et al., 2012
miR-200b	miRNA	down	IL-1β, IL-6, IL-8 and TNF-α	Clinical samples	Immune response	Isomoto et al., 2012
miR-200c	miRNA	down	IL-1β, IL-6, IL-8 and TNF-α	Clinical samples	Immune response	Isomoto et al., 2012
miR-223	miRNA	up	NF-kB and ARID1A	Clinical samples and cell lines	CagA Associated Gastric Carcinogenesis	Matsushima et al., 2011; Yang et al., 2018
miR-375	miRNA	down	IL-1β, IL-6, IL-8 and TNF-α	Clinical samples	Immune response	Isomoto et al., 2012
miR-429	miRNA	down	IL-1β, IL-6, IL-8 and TNF-α	Clinical samples	Immune response	Isomoto et al., 2012
miR-532	miRNA	down	IL-1β, IL-6, IL-8 and TNF-α	Clinical samples	Immune response	Isomoto et al., 2012
lncRNA AF147447	lncRNA	down	MUC2	Clinical samples	Cell proliferation and invasion	Zhou et al., 2016
n345630	lncRNA	down	undefined	Clinical samples and cell lines	undefined	Zhu et al., 2015
XLOC_004787	lncRNA	down	undefined	Clinical samples and cell lines	undefined	Zhu et al., 2015
n378726	lncRNA	down	undefined	Clinical samples and cell lines	undefined	Zhu et al., 2015
LINC00473	lncRNA	down	undefined	Clinical samples and cell lines	undefined	Zhu et al., 2015
XLOC_005517	lncRNA	up	undefined	Cell lines	undefined	Zhu et al., 2015
LINC00152	lncRNA	up	undefined	Cell lines	undefined	Zhu et al., 2015
XLOC_13370	lncRNA	up	undefined	Cell lines	undefined	Zhu et al., 2015
n408024	lncRNA	up	undefined	Cell lines	undefined	Zhu et al., 2015
XLOC_004122	lncRNA	down	undefined	Clinical samples and cell lines	undefined	Yang et al., 2015
XLOC_014388	lncRNA	down	undefined	Clinical samples and cell lines	undefined	Yang et al., 2015
XLOC_004562	lncRNA	up	undefined	Cell lines	undefined	Yang et al., 2015
XLCO_005912	lncRNA	up	undefined	Cell lines	undefined	Yang et al., 2015
XLCO_000620	lncRNA	up	undefined	Cell lines	undefined	Yang et al., 2015

### Host miRNAs associated with *H. pylori* infection

In this section, we focus on the role of miRNAs at the early stages of the pre-neoplastic process of GC since, as mentioned above, it is at these stages where *H. pylori* is directly involved (Chen et al., 2016). We do not make an in-depth discussion of miRNAs that are differentially expressed in GC as, at this stage, *H. pylori* is most commonly not present at the lesions.

miRNAs have an important role in the control of inflammatory response associated with *H. pylori* infection. Downregulation of let-7 miRNAs, the first known human miRNA family, was observed in *H. pylori*-positive clinical samples, particularly those infected with CagA-positive strains. Downregulation of let-7b results in overexpression of TLR4, which activates NF-κB and increases the expression of COX-2 (Teng et al., 2013). Similarly, members of the miR-200 family, including miR-141, miR-200a/b/c, and miR429, as well as miR-375 and miR-103, were downregulated in *H. pylori*-infected gastritis samples. These findings correlate with increased neutrophil and/or mononuclear cell tissue infiltration, as well as overexpression of pro-inflammatory cytokines including IL-1β, IL-6, IL-8, and TNF-α (Isomoto et al., 2012). miR-124 has tumor suppressive functions and was reported as a negative regulator of polyamine catabolic enzyme spermine oxidase (SMOX) (Murray-Stewart et al., 2016). SMOX is induced in bacterial infection and chronic inflammation, including *H. pylori*-associated gastritis. When induced in the nucleus, SMOX-dependent production of hydrogen peroxide contributes to oxidative DNA damage and subsequent tumorigenesis. Consequently, chronic gastritis patients with lower levels of miR-124 expression displayed higher levels of SMOX and were more likely to develop high-grade gastric lesions.

On the other hand, several miRNAs are upregulated in *H. pylori* infection. miR-21 was the first reported miRNA to be influenced by *H. pylori*, found overexpressed in *H. pylori*-infected cell lines, gastritis, and GC tissues (Zhang et al., 2008). Importantly, miR-21 acts as an oncogenic miRNA by targeting the tumor suppressor gene RECK, involved in tumor metastasis and angiogenesis (Gutiérrez et al., 2016). miR-155 expression is induced in *H. pylori*-infected GES-1, as well as in gastritis tissue samples (Xiao et al., 2009). Interestingly, expression of this miRNA is induced in *H. pylori*-infected T cells and primary macrophages in a Foxp3-dependent manner, suggesting a functional interplay between miR-155 and the host immune response (Fassi Fehri et al., 2010). miR-155 also targets IkB kinase-ε (IKK-ε), Sma- and Mad-related protein 2 (SMAD2), and Fas-associated death domain protein (FADD), reducing the release of pro-inflammatory cytokines such as IL-8 and GROα (CXCL1) and attenuating the inflammatory response associated with *H. pylori* infection (Xiao et al., 2009). miR-223 is also induced upon *H. pylori* infection (Matsushima et al., 2011; Wang et al., 2016). This miRNA downregulates pro-inflammatory cytokines (IL-6, IL-8, IL-12, and TNF-α) and inhibits the activation of macrophages through downregulation of CD40, CD68, CD80, and CD163. Therefore, miR-223 also limits the host inflammatory response to *H. pylori* (Wang et al., 2016). Additionally, CagA-positive *H. pylori* strains induce expression of NF-κB, which binds to the promoter of miR-223. miR-223 targets ARID1A, promoting cell proliferation and migration. In clinical samples miR-223 is upregulated in the tumor, while ARID1A is downregulated significantly in comparison to the non-tumor adjacent mucosa from the same patients (Yang et al., 2018).

Taken together, host miRNAs play a preponderant role in the inflammatory response against *H. pylori*, representing a potential bridge between *H. pylori*, chronic inflammation and progression of precancerous lesions. Table 1 summarizes the main studies of host miRNAs which are dysregulated upon infection by *H. pylori*.

### EBV ncRNAs and miRNAs

In latent EBV infections, the virus hinders the host functional immunity. During latent infection, EBV expresses limited levels of protein but high levels of ncRNAs, such as Epstein-Barr Virus-Encoded RNAs (EBERs) and Bam HI A rightward transcripts (BARTs). This suggests that the ncRNAs are related to evasion of the viral immune response (Albanese and Tagawa, 2017). Pfeffer, were the first group to report that miRNAs are also encoded by viruses (Pfeffer et al., 2004). The herpesvirus family encodes the vast majority of viral miRNAs (Pfeffer et al., 2005). These DNA viruses, including EBV, encode their own miRNAs and/or modulate the expression of host miRNAs to facilitate infection cycles. It has also been evidenced that EBV miRNAs may contribute to gastric carcinogenesis (Yau et al., 2014). EBV miRNAs are encoded in two main clusters: the Bam HI fragment H rightward open reading frame 1 (BHRF1) cluster and BART cluster (Pfeffer et al., 2004).

### ncRNAs EBERs and BARTs

It is long known that the most abundantly expressed viral transcripts during EBV latent infection are small non-coding EBV-encoded RNAs termed EBER1 and EBER2 (Rymo, 1979). These ncRNAs are 167 and 172 nucleotides long, respectively (Lerner et al., 1981), and form double-stranded RNA-like structures. The genes encoding EBER1 and EBER2 are separated by 161 nucleotides and transcribed individually in the same direction using RNA polymerase III (Rosa et al., 1981). These transcripts are expressed in all EBV-positive cells and, thus, in all EBV-associated malignancies. EBERs can be detected in EBV-positive cells using *in situ* hybridization technique as a routine test to determine EBV infection in clinical samples (Weiss and Chen, 2013).

In the host, EBERs are involved in the promotion of cellular growth. Reports show that EBERs may confer an apoptosis-resistant phenotype to epithelial cells. EBV infection induces expression of insulin-like growth factor (IGF-I) and secreted IGF-I acts as an autocrine growth factor. Moreover, EBV-positive GC and NPC biopsies consistently express IGF-I, whereas EBV-negative biopsies do not (Iwakiri et al., 2003, 2005).

BARTs are a family of transcripts from the Bam HI A region of the EBV genome. BARTs show extraordinarily high expression levels in EBV-infected epithelial cancers, but not in EBV-transformed lymphocytes (Qiu et al., 2011). It has been proposed that BARTs are key players in epithelial malignancies such as NPC and EBVaGC. Reports indicate that the expression of BARTs is regulated by interferon regulatory factors and NF-κB signaling pathway (Chen et al., 2005; Samanta et al., 2006). Interest in the functional role of BARTs has led to the discovery of viral miRNAs (miR-BARTs) in this region (Pfeffer et al., 2004).

### EBV miRNAs

EBV was the first described virus to encode miRNAs (Pfeffer et al., 2004). EBV uses the host cell machinery to produce its miRNAs, analogous to the biogenesis of host cell miRNAs (Kim and Lee, 2012). Briefly, the miRNA sequences are mainly transcribed by host RNA polymerase II to form stem-loop structures, which are subsequently processed by Drosha and DiGeorge syndrome chromosomal region 8 (DGCR8) and Dicer. The host DGCR8 endonuclease complex cleaves the primary miRNA, resulting in a precursor miRNA which is transported from the nucleus to the cytoplasm. Subsequently, this precursor miRNA is processed in the cytoplasm by host protein Dicer to generate the mature miRNA.

As stated above, EBV miRNAs are encoded in two clusters. The first EBV miRNA cluster is located within a transcript that encodes the BHRF1 protein and includes three miRNA precursors (miR-BHRF1−1,−2, and−3), which produce four mature EBV miRNAs. miR-BHRF1-1 is located in the 5′ UTR (untranslated region) while miR-BHRF1−2 and−3 are located in the 3′ UTR of the BHRF1 mRNA (Pfeffer et al., 2004). The second cluster is located in intronic segments within the BART transcripts and includes 22 miRNA precursors (miR-BART1-22), which generate 40 mature miRNAs (Cai et al., 2006; Grundhoff et al., 2006; Zhu et al., 2009).

The expression of these microRNAs depends on the latency state and is cell-type specific. miR-BHRF1s are expressed during lytic infection and in latency III (Cai et al., 2006). Meanwhile, miR-BARTs are expressed in all EBV latency types. miR-BHRF1s are expressed in lymphoblastoid cell lines (latency III), but not in NPC (Cosmopoulos et al., 2009) and EBVaGC (Kim et al., 2007; Qiu et al., 2011; Shinozaki-Ushiku et al., 2015). Only sporadic expression of BHRF1s at very low levels was found in some Burkitt's lymphoma and Hodgkin's disease biopsies (Qiu et al., 2011). Conversely, miR-BARTs are expressed in EBV-infected lymphoblastoid cell lines, Burkitt's lymphoma, NPC and EBVaGC, showing significantly higher overall expression in epithelial cancers NPC and EBVaGC in comparison to EBV-positive B lymphoma. As expression of BHRF1 miRNAs is not consistently deregulated in the tumors, it is expected that the oncogenic role of EBV miRNAs can be attributed to the BARTs (Qiu et al., 2011). Specific miR-BARTs can induce transition between latency states and lytic replication. miR-BART6-5p maintains type I/II latency in cultured cells, as its inhibition results in increased expression of viral oncoproteins latent membrane protein 1 (LMP1) and EBNA2, important in type III latency (Iizasa et al., 2010).

EBV miRNAs favor evasion of the host immune system and maintain EBV latency by suppressing apoptosis of infected cells (Seto et al., 2010; Albanese and Tagawa, 2017). Viral miRNAs can regulate inflammation by targeting several chemokines and cytokines that regulate antiviral inflammatory responses. For example, several viral miRNAs can act collectively to suppress the release of pro-inflammatory cytokine IL-12 caused by and subsequently modulate the inflammatory response of CD4+ and CD8+ T cells (Albanese et al., 2016; Tagawa et al., 2016). EBV miRNAs can also target several viral genes to downregulate expression of some EBV proteins such as EBNA1 (Albanese et al., 2016), latent membrane proteins LMP1 (Lo et al., 2007) or LMP2A (Lung et al., 2009). Very low expression of viral antigens allows the virus to escape the immune system and maintain persistent infection.

Similarly, Marquitz and colleagues (Marquitz et al., 2012) demonstrated that EBV infected GC cell line shows limited viral protein expression whereas viral BART miRNAs are abundantly expressed. Moreover, BARTs affected growth properties of these EBV-infected cells, suggesting that they can contribute to the development of epithelial malignancies. In an another study they showed that both BART clusters can independently inhibit apoptosis in GC cell line and that the pro-apoptotic protein Bcl-2 interacting mediator of cell death is a target of multiple BART miRNAs (Marquitz et al., 2011). These observations were further extended by identification of several mRNA targets for EBV BART miRNAs that encode pro-apoptotic proteins (CASZ1, DICE1, OCT1, CREBBP, SH2B3, PAK2, and TP53INP1; Kang et al., 2015).

Specifically, the EBV miR-BART5 is highly expressed in epithelial cells. This miR-BART targets the proapoptotic protein p53 up-regulated modulator of apoptosis (PUMA) mRNA, resulting in translational repression of the protein in NPC and EBV-infected GC cells and promoting host cell survival (Choy et al., 2008). miR-BART20-5p reduces apoptosis and enhances growth of GC cells by targeting apoptosis-inducing factor Bcl-2-associated agonist of cell death (BAD) mRNA (Kim et al., 2015). Recently, high BART20-5p expression levels were associated with worse recurrence-free survival of EBVaGC patients (Kang et al., 2017). Likewise, miR-BART4-5p targets BH3 interacting domain death agonist (BID), a Bcl-2 family gene, and suppresses apoptosis in GC cells (Shinozaki-Ushiku et al., 2015).

Consequently, miR-BARTs play an important role in carcinogenesis. It was reported that several EBV-encoded miRNAs, including miR-BART22, contribute to EBVaGC by targeting N-myc downstream regulated gene 1 (NDRG1) (Kanda et al., 2015). NDRG1 is an essential gene for maintaining epithelial cell differentiation and a suppressor of metastasis. miR-BART11 was shown to promote monocyte differentiation to macrophages by attenuating expression of forkhead box P1 (FOXP1), a key molecule involved in this transformation (Song et al., 2016). Biopsies from both epithelial cancers, NPC (Liao et al., 2014) and GC (Liu et al., 2017), are often infiltrated with inflammatory cells, including tumor-associated macrophages that serve as a barrier against the infiltration of CD8+ T cells into GC. In the case of NPC, tumor infiltration by macrophages is tightly associated with poor prognosis (Liao et al., 2014). Targeting of FOXP1 by miR-BART11 induces proliferation of GC cells and activates NF-κB signaling (Song et al., 2016). In a study which analyzed 52 EBVaGC tissues samples for viral miRNAs, miR-BART4-5p was the most abundantly expressed miR-BART, followed by miR-BART11-3p, miR-BART2-5p, miR-BART6-3p, miR-BART9-3p, and miR-BART18-5p. Additionally, an interaction among miR-BART9-3p, miR-200a and E-cadherin was demonstrated suggesting that miR-BARTs contribute to malignant transformation via regulation of host miRNAs and epithelial-to-mesenchymal transition (EMT) (Tsai et al., 2017). Other EBV BART-miRNAs, including miR-BART1-3p, 5-5p, 7-3p, 15-3p, 19-3p, and 22-3p are also expressed in EBVaGC tissue samples and GC cell lines (Qiu et al., 2011; Kim et al., 2013), and have distinct expression levels in EBV-related epithelial cancers compared to lymphoid malignancies. The functions of these and additional miR-BARTs are included in Table 2.

**Table 2:** EBV miR-BARTs in EBV infected cells or tissues.

**Cluster**	**miRNA**	**Target**	**Target gene function**	**miRNA effect**	**Number of patients**	***In vitro* model**	**References**
1	BART1			Overexpressed in GC	59 GC	no	Kang et al., 2017
1	BART3	IPO7	Nuclear importer protein	Immune evasion	no	Human B cell line	Dölken et al., 2010
1	BART3	DICE1	Tumor suppressor	Increased cell proliferation	11 NPC	Hela, Hek, GC	Lei et al., 2013
1	BART4	BID	Pro-apoptotic protein	Anti-apoptotic in GC cell lines	10 GC	GC	Shinozaki-Ushiku et al., 2015
1	BART4			Overexpressed in GC	59 GC	no	Kang et al., 2017
1	BART5	PUMA	Pro-apoptotic protein	Inhibition of apoptosis	15 NPC	HeLa, HEK, NPC, GC	(Choy et al., 2008)
1	BART6-5p	DICER	miRNA biogenesis	Unknown	no	LCL, BL, Hek, HeLa	Iizasa et al., 2010
1	BART6-3p	LOC553103	Unknown	Reduced migration and invasion	no	NPC, GC	He et al., 2016
1	BART15-3p	BRUCE	Anti-apoptotic	Increased apoptosis	no	GC	Choi et al., 2013
1	BART15	NLPR3	Regulation of inflammation	Immune evasion	no	Macrophages	Haneklaus et al., 2012
1	BART16	TOMM22	Mitochondrial receptor for pro-apoptotic protein BAX	Inhibition of apoptosis	no	Human B cells line	Dölken et al., 2010
1	BART1-3p + BART16	CASP3	Pro-apoptotic	Inhibition of apoptosis	no	BL	Vereide et al., 2013
1	BART3 + BART16	IPO7	Nuclear importer protein	Immune evasion	no	BL	Vereide et al., 2013
2	BART9	CDH1		Increased proliferative and invasion activity in SNU719	1039 GC	GC	Tsai et al., 2017
2	BART11	FOXP1		Promotion of inflammation	no	NPC, GC, MONOCYTES	Song et al., 2016
2	BART13-3p	CAPRIN2	Wnt-signaling	Inhibition of apoptosis	no	BL	Riley et al., 2012
2	BART19-3p	WIF1	Wnt inhibitor	Deregulation of the canonical Wnt-signaling pathway	20 NPC	NPC	Wong et al., 2012
2	BART19-3p + BART14 + BART18	NLK	Wnt Inhibitor	Deregulation of the canonical Wnt-signaling pathway	20 NPC	NPC	Wong et al., 2012
2	BART19-3p + BART7 + BART17	APC	Wnt Inhibitor	Deregulation of the canonical Wnt-signaling pathway	20 NPC	NPC	Wong et al., 2012
2	BART20	BZLF1, BRLF1^*^	Viral proteins		no	GC	Jung et al., 2014
2	BART20			Associated to poor survival	59 GC	no	Kang et al., 2017
2	BART20	BAD	Apoptosis regulator	Inhibition of apoptosis and suppression of lytic induction	no	GC	Kim et al., 2015
	BART2	BALF5^*^	Viral polymerase	Inhibition of transition from latent to lytic viral replication	no	Hela, BL	Barth et al., 2007
	BART2	MICB	NK cell ligand	Immune evasion	no	HEK,CC, HeLa, LL	Nachmani et al., 2009

* *Viral protein. LCL, lymphoblastoid cell line; BL, Burkitt's lymphoma; GC, gastric cancer; NPC, nasopharyngeal cancer; HEK, Human embryonic kidney cell line; CC, colon carcinoma; LL, lymphoma cell line*.

Contrary to most published studies, a few publications bring controversial view on the strictly pro-tumorigenic role of miR-BARTs. For example, it has also been proposed that miR-BART6-3p acts as a tumor suppressor miRNA. miR-BART6-3p directly targets lncRNA LOC553103 and induces downregulation of CDH2, β-catenin, SNAIL, MMP2, and MMP9, reversing EMT (He et al., 2016). Choi et al. (2013) have shown that miR-BART15-3p, expressed in cultured GC cells infected with EBV, targets baculovirus inhibitor of apoptosis repeat-containing ubiquitin-conjugating enzyme (BRUCE), a member of the inhibitor of apoptosis proteins. These findings show that some miR-BARTs can possess anti-tumor activities that have, to date, been poorly described.

### Host miRNAs associated with EBV infection

EBV modulates the host immune response and participates in cell transformation not only via its own miRNAs but also through regulation of host miRNAs. In this sense, several studies have investigated the expression of viral and host miRNAs in EBVaGC. For example, Iizasa et al. (2010) reported that viral miR-BART6-5p targets host cell Dicer by direct binding to the 3′-UTR of the mRNA, and proposed it is likely a major viral miRNA suppressor. In consequence, host cell miRNA production is impaired, thus enabling EBV to remain in a specific latency state and evade the host immune system.

Through a sequential series of studies, the group of Fukayama showed that expression the miR-200 family was decreased in EBVaGC, as well as in several GC cell lines infected with EBV (Shinozaki et al., 2010). They also reported that Bam HI A fragment rightward reading frame (BARF)0, EBNA1, EBERs, and LMP2A contributed to the downregulation of the mature miR-200 family, indicating a synergetic effect of latency type I genes in EBVaGC. Importantly, *in vitro*, downregulation of miR-200 transcripts lead to upregulation of E-cadherin transcription repressors ZEB1 and ZEB2. In consequence, decreased expression of miR-200 resulted in inhibition of E-cadherin expression and induction of EMT, which was initially observed by their group to be more frequent in EBVaGC than in EBV-negative GC (Sudo et al., 2004). Confirming these findings, Marquitz et al. (2014) performed miRNA profiling of an EBV-infected GC cell line and reported the downregulation of several miRNAs from the miR-200 and let-7 family. Moreover, they identified several other downregulated miRNAs, among them miR-143-3p, miR-146b-5p, miR-148-3p. Another downregulated host miRNA in EBVaGC is miR-34a. Expression of this miRNA is inhibited by viral protein EBNA1 (Kim et al., 2017), which is expressed by all EBV-infected cells, and is an essential gene for the establishment and maintenance of latency (Ribeiro et al., 2017). Downregulation of miR-34a in EBV-infected GC cells causes upregulation of NOX2 and results in augmented ROS production and increased cell viability (Kim et al., 2017).

The recent molecular classifications of GC proposed by the TCGA, also identified a differential miRNA expression profile for EBVaGC. Upregulation of miR−196a and−196b was observed in all GC subtypes compared to normal tissue samples, except this subtype (The Cancer Genome Atlas Research Network, 2014). A study by Treece and collaborators (Treece et al., 2016) that analyzed expression of a custom miRNA panel in EBV-positive versus EBV-negative tumors showed significant downregulation of miR-196b in the infected tumors, confirming what was identified by the TCGA. The same study demonstrated significant upregulation of miR-155, miR-185, and miR-378 in EBV-positive tumors, also consistent with previously reported upregulation of miR-155 and miR-185 in the TCGA database. miR-155 is upregulated in both, in EBV-positive tumors (Jiang et al., 2006; Du et al., 2011) and in *H. pylori*-infected gastric tissues, and suppresses the host inflammatory response, as described above in the section about host miRNAs associated with *H. pylori* infection.

In summary, EBV-infected cells express viral miRNAs and proteins which regulate host miRNA expression. This helps infected cells to evade the host immune response, favoring chronic infection and enhancing cell viability.

## Exosomes

Exosomes are extracellular vesicles which originate from multivesicular bodies and span between 30 and 100 nm in diameter. Exosomes are secreted from healthy, cancer and virus-infected cells (Kowal et al., 2014). Recently, they have been recognized as a tool that can be used by cancer and virus-infected cells to manipulate their microenvironment, influencing the growth of neighboring cells through the intercellular transfer of various signaling molecules and viral miRNAs (Meckes et al., 2010). Viruses share several properties with exosomes. Their diameter ranges from 30 to 300 nm and they can also be released through the multivesicular pathway (Meckes and Raab-Traub, 2011; Raab-Traub and Dittmer, 2017). It has been also shown that oncogenic viruses can alter the content of exosomes, favoring persistent infection and pathogenesis.

### Exosomes and EBV infection

Compared with viruses, exosomes possess an increased ability to enter a wider range of cell types. *In vitro* experiments showed that when exposed sufficient time, most cell types internalized at least a part of stained exosomes independently of their cell origin (van Dongen et al., 2016). The exact mechanisms of exosome entry, specific cell targeting, and cargo delivery remains unclear. Generally, exosomes can deliver their content by fusing to the recipient cell's cytoplasmic membrane. Nevertheless, receptor-specific entry has also been described. Incorporation of viral glycoprotein gp350 has been reported in exosomes produced by EBV-infected cells. These exosomes selectively target B cells through the EBV entry receptor CD21. This gp350–CD21 interaction is so strong that exosomes derived from EBV-transformed cells can cause competitive inhibition of EBV entry in B cells, preventing their infection (Vallhov et al., 2011). In contrast, viral entry has been broadly studied and is very specific for each virus and cell type, requiring specific receptors and co-receptors that bind to envelope glycoproteins. How extracellular vesicles and exosomes exploit viral entry routes for cargo delivery and how viruses utilize exosomes for virus, viral genome or protein delivery has been comprehensively reviewed elsewhere (Nolte-'t Hoen et al., 2016; van Dongen et al., 2016; Raab-Traub and Dittmer, 2017).

A study by Pegtel et al. (2010) evidenced that EBV-infected B cells secrete functional viral miRNA miR-BHRF1-3 via exosomes. This miRNA is transferred to and acts by silencing expression of target genes in uninfected recipient cells. Furthermore, they also detected EBV-encoded BART Cluster 1 miRNAs in circulating non-B cell lymphocytes, where EBV DNA is not present. They suggest that the presence of miR-BARTs in these cells can be attributed to exosomal transfer. It is known that miRNAs from the miR-BART cluster 1 cause translational repression of EBV LMP1 (Lo et al., 2007). They propose exosomal miRNA transfer from EBV-infected cells to uninfected recipient cells as a gene silencing mechanism in adjacent (in a paracrine fashion) or distant cells. Accordingly, Choi et al. have described that miR-BART15-3p is secreted via exosomes by EBV-infected GC cells, targeting the inhibitor of apoptosis BRUCE (Choi et al., 2013). They also proposed that this miR-BART is preferentially loaded into exosomes and may be delivered into neighboring immune cells, favoring their apoptosis. Though interesting, no experiments were carried out to support this conclusion.

Aside from non-coding RNAs, exosomes derived from EBV-positive cells contain functional viral proteins. LMP1 is the major EBV oncoprotein, as it is required for B lymphocyte transformation, and is expressed in neoplastic cells of EBV-positive lymphoid malignancies (Raab-Traub and Dittmer, 2017). Dukers et al. (2000) have for the first time described direct immunosuppression in a DNA virus, previously thought to be restricted to RNA viruses. This group demonstrated that EBV is capable of inducing T cell anergy via a novel direct route, possibly mediated by secretion of EBV-encoded LMP1. EBV-infected cells secrete LMP1-containing exosomes, which inhibit T cell proliferation and natural killer cell cytotoxicity (Dukers et al., 2000; Flanagan et al., 2003); suggesting that these exosomes contribute to the immune suppression that allows the virus to survive. LMP1 also increases the upload and release of fibroblast growth factor 2 (FGF2), a potent angiogenic factor, into exosomes (Ceccarelli et al., 2007). Another study that investigated exosomes released from NPC cells harboring latent EBV (Meckes et al., 2010) showed that exosomes contained EBV-LMP1. Furthermore, uptake of these exosomes resulted in the activation of phosphoinositide 3-kinase (PI3K)/AKT and MAPK/ERK signaling pathways in the recipient cells. Exosomes loaded with LMP1 are associated with increased oncogenicity. How LMP1 enters or manipulates the host exosome pathway is still not clear. The group of Meckes first showed that the host tetraspanin protein CD63 regulates packing of LMP1 into exosomes, as well as LMP1-mediated enhancement of vesicle production, exosomal trafficking, noncanonical NF-κB signaling (Hurwitz et al., 2018), and mTOR signaling (Hurwitz et al., 2017). Next, they investigated the interactome of LMP1 where, among the probable interacting partners of LMP1, they identified components of exosomes or other extracellular vesicles, including molecules involved in vesicular trafficking, such as CD63, syntenin-1, ALIX, TSG101, and HRS. Based on these results, they conclude that LMP1 likely modifies pathways involved in exosome trafficking and biogenesis (Rider et al., 2018).

Although LMP1 plays a crucial role in EBV-mediated malignancies, LMP1 has seldom been detected in EBV-positive gastric tumors. A study by Sato et al. (2017) provides a hint toward the mechanism explaining this controversial observation. They demonstrated that co-culture of LMP1-positive and -negative gastric cancer cells leads to elimination of LMP1-positive cells, through the release of LMP1-loaded exosomes that mediate epidermal growth factor receptor (EGFR) activation in LMP1-negative cells. Assuming that the microenvironment and neighboring cells can also influence viral latency eliminating cells expressing the viral latent gene LMP1 during the early phase of EBV infection of gastric cells, LMP1-negative cells gradually overgrow and replace LMP1-positive cells.

EGFR can be secreted from cells into exosomes and other microvesicles. Subsequent uptake of these EGFR-enriched exosomes by endothelial cells induces activation of MAPK and Akt pathways, and triggers endogenous expression of vascular endothelial growth factor (VEGF), followed by the activation of VEGF receptor-2 (VEGFR-2) (Al-Nedawi et al., 2009). After exosome internalization in an EBV-negative epithelial cell line, exosome-derived LMP1 induced expression of EGFR. Importantly, these non-infected cells produced exosomes that also contained high levels of EGFR (Meckes et al., 2010). In a similar fashion, LMP1 activates expression of hypoxia-inducible factor-1α (HIF1α), a transcriptional regulator under hypoxic conditions that promotes a more aggressive tumor phenotype. LMP1 increases exosome delivery of transcriptionally active HIF1α (Aga et al., 2014). These findings show that, via exosome-mediated transfer, HIF1α promotes pro-metastatic effects in recipient cells.

**Figure 2:**
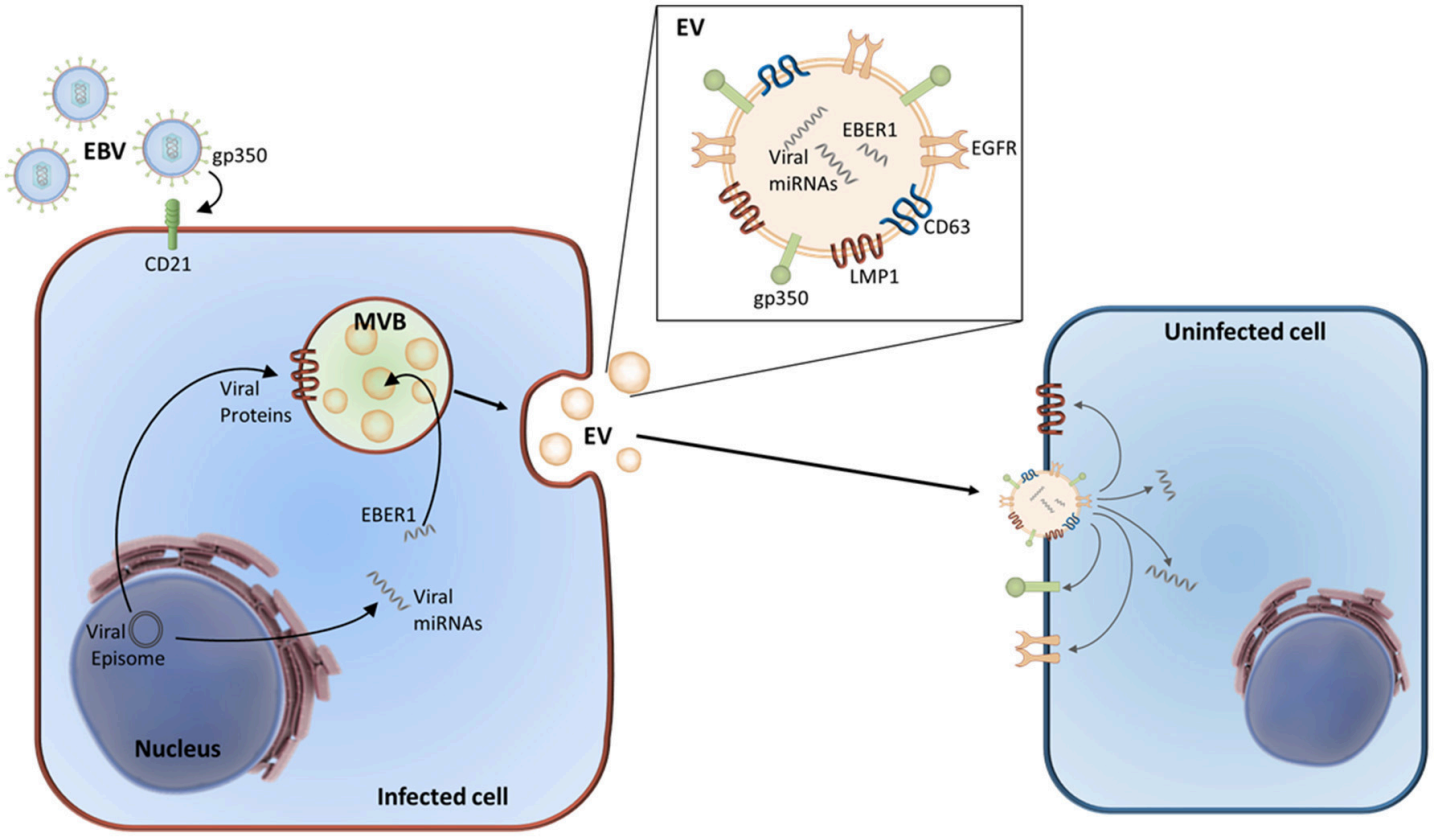
Exosome remodeling by EBV. EBV enters in the cell via the recognition of gp350 by the CD21 receptor. EBV-infected cells produce exosomes enriched for LMP1, gp350, and EGFR. Alongside cellular RNAs, these exosomes also contain viral RNAs, miRNAs, and proteins. Following the release of these EBV-modified exosomes, they can enter uninfected cells where their cargo can be incorporated into the recipient cell and start their mechanism of action.

In summary, exosomes from EBV-infected cells have been shown to incorporate a wide range of molecules such as LMP1, EGFR, FGF2, PI3K, and HIF1α, as well as multiple kinases (Figure 2). Taken together, these observations suggest that the functional properties of exosomes derived from EBV-infected cells mimic the properties of LMP1-expressing cells and thus, possibly regulate signaling pathways in both an autocrine and paracrine manner (Meckes et al., 2013; Meckes, 2015; Raab-Traub and Dittmer, 2017).

### Exosomes and *H. pylori* infection

Secretion of CagA oncoprotein can induce malignant neoplasms in mammals and is strongly associated with severe gastric lesions, particularly GC. In host cells, CagA affects multiple signaling pathways by acting as an extrinsic scaffold protein (Hatakeyama, 2014; Yong et al., 2015). CagA-positive *H. pylori* infection may also be associated with diseases outside of the stomach (Franceschi and Gasbarrini, 2007).

To date, only one study (Shimoda et al., 2016) has investigated whether host exosomes may be modified by *H. pylori* in a manner similar to that in which viruses hijack host exosomes. This study reported that serum-derived exosomes in patients infected with CagA-positive *H. pylori* were also loaded with CagA. When CagA expression was induced in gastric epithelial cells these cells also secreted exosomes containing CagA. Additionally, it was demonstrated that exosomes deliver functional CagA into gastric epithelial cells, as they were able to induce elongated cell shape in these cells. These findings indicate that CagA can be delivered to distant organs or tissues via secretion of CagA-enriched exosomes into circulation. Thus, these exosomes may be involved in the development of extra-gastric diseases associated with CagA-positive *H. pylori* infection. Furthermore, exosome-mediated CagA delivery may contribute to the increased risk of colorectal (Strofilas et al., 2012) and pancreatic (Risch et al., 2014) cancers in individuals infected with CagA-positive *H. pylori* strains.

## Bacterial outer membrane vesicles (OMVs)

So far, little is known about exosomes produced by cells under *H. pylori* infection, though it has been shown that *H. pylori* itself produces extracellular vesicles that can be seen as bacterial-like exosomes. Several bacterial species including *H. pylori* (Fiocca et al., 1999) have been reported to produce OMVs, both *in vivo* and *in vitro*. OMVs are small spheres of size between 20 and 300 nm consisting of phospholipid bilayers that are released into the extracellular medium of the outer membrane of Gram-negative bacteria (Beveridge, 1999). To a certain degree, OMVs share many similarities with exosomes, though their biogenesis is different as they are formed by budding from the bacterial surface. OMVs contain outer membrane and periplasmic constituents such as lipopolysaccharide, peptidoglycan, phospholipids, outer membrane and cytoplasmic proteins, nucleic acids, ion metabolites, and signaling molecules (Parker and Keenan, 2012). OMVs of pathogenic bacteria are enriched with specific proteins which favor their invasiveness (Jan, 2017). The production and content of OMVs can vary during different growth stages and conditions. OMVs do not only play role in bacterial communication, where they can even transfer antibiotic resistance (Chattopadhyay and Jaganandham, 2015), but also in host-pathogen interaction where they access the bloodstream and tissues. Secreted OMVs transfer biological molecules such as virulence factors, toxins, adhesion molecules, and other immunomodulatory compounds to the host cell and, accordingly, enhance bacterial survival inside the host (Jan, 2017).

Olofsson et al. (2010) performed a detailed biochemical and functional characterization of *H. pylori*-derived vesicles. Using mass spectrometry, more than 300 different *H. pylori* proteins were identified in these vesicles—among them virulence factors CagA and VacA, adhesins BabA and SabA, urease subunits and gamma-glutamyltransferase (GGT). These molecules are well known to contribute to *H. pylori* virulence and colonization. In this study, they also demonstrated that CagA is present on the surface of OMVs, showing that CagA is delivered to gastric host cells via *H. pylori* vesicles. VacA is secreted in OMVs and, after delivery, modulates pathological activity in host cells (Ricci et al., 2005). Moreover, if the urease subunits were active, VacA could decrease pH, facilitating *H. pylori* colonization (Parker and Keenan, 2012).

GGT is a virulence factor that contributes to cell-cycle arrest, apoptosis, and necrosis in gastric epithelial cells. Additionally, through the inhibition of T cell-mediated immunity and dendritic cell differentiation, GGT induces immune tolerance, favoring persistent infection and gastric colonization by *H. pylori* (Ricci et al., 2014). GGT secreted in OMVs can be delivered to resident lymphocytes within the lamina propria of the gastric mucosa. Therefore, GGT can inhibit lymphocyte proliferation, facilitating bacterial invasion and growth (Zhang et al., 2013). Several malignancies express active GGT, which was also shown to be released from cancer cells in association with exosome-like vesicles (Franzini et al., 2014).

*H. pylori* OMVs suppress human T cell response indirectly, via induction of COX-2 expression in monocytes (Hock et al., 2017). Similarly, COX-2 activity may have a broader modulatory effect on the immune response. COX-2 is responsible for generation of prostaglandins and is induced during inflammation. *H. pylori* OMVs also induce expression of immunosuppressive cytokines IL-10 and IL-6 in human peripheral blood mononuclear cells, as well as induction of apoptosis of T cells (Winter et al., 2014).

The link between OMVs and gastric carcinogenesis remains unknown. Yet, recent evidence shows that *H. pylori* OMVs induce the surface expression of intercellular adhesion molecule 1 (ICAM-1) in primary gastric epithelial cells (Ko et al., 2015). Interestingly, about 50% of GC tissues overexpress ICAM-1 which correlates with increased metastatic potential and poorer prognosis (Jung et al., 2012).

In summary, OMVs allow *H. pylori* to modulate the innate inflammatory response by limiting inflammation and aiding in the evasion of the immune response. In addition, they enable distant delivery of specific virulence factors to host cells without direct cell interactions. However, the presence of OMVs in the infected host blood has not yet been demonstrated (Shimoda et al., 2016). This is a promising field that can provide novel insights into *H. pylori* pathogenesis.

## Conclusions

Deregulation of ncRNAs, including lncRNAs and miRNAs, plays a pivotal role in carcinogenesis. In GC, two ethiologic agents, *H. pylori* and EBV, contribute to the deregulation of these ncRNAs. Both pathogens have evolved mechanisms that suppress the host inflammatory response and enable chronic colonization of the stomach, while inducing inflammation that drives oncogenic modifications.

*H. pylori* and EBV alter the expression of host miRNAs which control inflammatory processes that favor immune evasion, contributing to bacterial growth or inhibition of apoptosis in EBV-positive cells. *H. pylori* and EBV can simultaneously downregulate the let-7 and miR-200 families, resulting in increased inflammation and induction of EMT, as well as increase the expression of miR-155, which suppresses the release of pro-inflammatory cytokines. The crosstalk between the pro- and anti-inflammatory miRNAs induced or suppressed by these pathogens results in the maintenance of persistent infection, while providing a microenvironment that drives oncogenic transformation of epithelial cells.

Individually, infection by *H. pylori* also promotes aberrant expression of lncRNAs, while EBV encodes its own viral ncRNAs and miRNAs. EBERs, BARTs, miR-BHRF1s, and miR-BARTs are expressed in EBV-infected cells. Overexpression of viral miRNAs together with downregulation of viral proteins are crucial for the maintenance of viral latency and evasion of the immune response, as well as inhibition of apoptosis of infected cells. EBV proteins expressed in the latent stage are capable of modifying the expression of host miRNAs. Finally, EBV miR-BART6-3p directly targets host Dicer, resulting in suppressed host miRNA production.

*H. pylori* strains differentially contribute to the progression of pre-neoplastic lesions through the dysregulation of miRNAs which promote a pro-inflammatory environment. CagA-positive strains induce enhanced inflammation and are strongly associated with GC. CagA can interfere with multiple host signaling pathways and promote carcinogenesis. Aside from type IV secretion, CagA is delivered to the host via extracellular vesicles, including host cell exosomes and bacterial OMVs. Upon delivery, CagA not only serves as a pathogenic scaffold, but can also inhibit miRNA let-7b causing the activation of inflammatory response mechanisms.

Exosomes and viruses, particularly retroviruses, share many physical and chemical characteristics, including their biogenesis. In addition, virus-infected cells produce exosomes with incorporated viral proteins and fragments of viral RNA. EBV-infected B cells have been shown to secrete exosomes enriched for EBV-miRNAs. These viral miRNAs are functionally active after delivery and also present in circulating non-infected non-B cells, suggesting that exosomes may have an important role in viral miRNA delivery *in vivo*. In summary, these findings show that EBV can modulate the exosomal cargo and use exosomes as a vehicle in communication with non-infected cells.

Uncovering the underlying role of miRNAs and lncRNA, as well as exosomes and OMV from infectious agents, in the precancerous gastric process is pivotal for a better understanding of the pathogenesis of GC.

## Author contributions

IP, SJ, IW, AS-B, NC-V, and AC: Drafted the manuscript. SJ and AS-B: Drafted the tables. IP, SJ, and IW: Drafted the figures. IP, IW, SJ, and AC: Critically revised the article. All authors read and approved the final article.

### Conflict of interest statement

The authors declare that the research was conducted in the absence of any commercial or financial relationships that could be construed as a potential conflict of interest.

## References

[B1] Aga M. Bentz G. L. Raffa S. Torrisi M. R. Kondo S. Wakisaka N.. (2014). Exosomal HIF1α supports invasive potential of nasopharyngeal carcinoma-associated LMP1-positive exosomes. Oncogene 33, 4613–4622. 10.1038/onc.2014.66 24662828 PMC4162459

[B2] Ajani J. A. Lee J. Sano T. Janjigian Y. Y. Fan D. Song S. (2017). Gastric adenocarcinoma. Nat. Rev. Dis. Primers 3:17036. 10.1038/nrdp.2017.36 28569272

[B3] Alarcón A. Figueroa U. Espinoza B. Sandoval A. Carrasco-Aviño G. Aguayo F. R. (2017). Epstein-barr virus–associated gastric carcinoma: the americas' perspective, in Gastric Cancer, eds Mozsik G. Karádi O. (InTech), 10.5772/intechopen.70201 Available online at: https://mts.intechopen.com/books/gastric-cancer/epstein-barr-virus-associated-gastric-carcinoma-the-americas-perspective

[B4] Albanese M. Tagawa T. (2017). MicroRNAs of Epstein-Barr virus control innate and adaptive antiviral immunity. J. Virol. 91:e01667–16. 10.1128/JVI.01667-16 28592533 PMC5533892

[B5] Albanese M. Tagawa T. Bouvet M. Maliqi L. Lutter D. Hoser J.. (2016). Epstein-Barr virus microRNAs reduce immune surveillance by virus-specific CD8+ T cells. Proc. Natl. Acad. Sci. U.S.A. 113, E6467–E6475. 10.1073/pnas.1605884113 27698133 PMC5081573

[B6] Al-Nedawi K. Meehan B. Kerbel R. S. Allison A. C. Rak J. (2009). Endothelial expression of autocrine VEGF upon the uptake of tumor-derived microvesicles containing oncogenic EGFR. Proc. Natl. Acad. Sci. U.S.A. 106, 3794–3799. 10.1073/pnas.0804543106 19234131 PMC2656159

[B7] Ambros V. (2004). The functions of animal microRNAs. Nature 431, 350–355. 10.1038/nature02871 15372042

[B8] Barth S. Pfuhl T. Mamiani A. Ehses C. Roemer K. Kremmer E.. (2007). Epstein–Barr virus-encoded microRNA miR-BART2 down-regulates the viral DNA polymerase BALF5. Nucleic Acids Res. 36, 666–675. 10.1093/nar/gkm1080 18073197 PMC2241876

[B9] Beveridge T. J. (1999). Structures of gram-negative cell walls and their derived membrane vesicles. J. Bacteriol. 181, 4725–4733. 10438737 10.1128/jb.181.16.4725-4733.1999 PMC93954

[B10] Byun E. Park B. Lim J. W. Kim H. (2016). Activation of NF-κB and AP-1 mediates hyperproliferation by inducing β-Catenin and c-Myc in Helicobacter pylori-infected gastric epithelial cells. Yonsei Med. J. 57, 647–651. 10.3349/ymj.2016.57.3.647 26996564 PMC4800354

[B11] Cai X. Schäfer A. Lu S. Bilello J. P. Desrosiers R. C. Edwards R.. (2006). Epstein-Barr virus microRNAs are evolutionarily conserved and differentially expressed. PLoS Pathog. 2:e23. 10.1371/journal.ppat.0020023 16557291 PMC1409806

[B12] Camargo M. C. Kim W. H. Chiaravalli A. M. Kim K. M. Corvalan A. H. Matsuo K.. (2014). Improved survival of gastric cancer with tumour Epstein-Barr virus positivity: an international pooled analysis. Gut 63, 236–243. 10.1136/gutjnl-2013-304531 23580779 PMC4384434

[B13] Camargo M. C. Murphy G. Koriyama C. Pfeiffer R. M. Kim W. H. Herrera-Goepfert R.. (2011). Determinants of Epstein-Barr virus-positive gastric cancer: an international pooled analysis. Br. J. Cancer 105, 38–43. 10.1038/bjc.2011.215 21654677 PMC3137422

[B14] Cardenas-Mondragon M. G. Carreon-Talavera R. Camorlinga-Ponce M. Gomez-Delgado A. Torres J. Fuentes-Panana E. M. (2013). Epstein Barr virus and *Helicobacter pylori* co-infection are positively associated with severe gastritis in pediatric patients. PLoS ONE 8:e62850 10.1371/journal.pone.0062850 23638154 PMC3634751

[B15] Cárdenas-Mondragón M. G. Torres J. Flores-Luna L. Camorlinga-Ponce M. Carreón-Talavera R. Gomez-Delgado A.. (2015). Case-control study of Epstein-Barr virus and *Helicobacter pylori* serology in Latin American patients with gastric disease. Br. J. Cancer 112, 1866–1873. 10.1038/bjc.2015.175 25996206 PMC4580389

[B16] Carrasco G. Corvalan A. H. (2013). *Helicobacter pylori*-induced chronic gastritis and assessing risks for gastric cancer. Gastroenterol. Res. Pract. 2013:393015. 10.1155/2013/393015 23983680 PMC3745848

[B17] Carrasco-Avino G. Riquelme I. Padilla O. Villaseca M. Aguayo F. R. Corvalan A. H. (2017). The conundrum of the Epstein-Barr virus-associated gastric carcinoma in the Americas. Oncotarget 8, 75687–75698. 10.18632/oncotarget.18497 29088902 PMC5650457

[B18] Ceccarelli S. Visco V. Raffa S. Wakisaka N. Pagano J. S. Torrisi M. R. (2007). Epstein-Barr virus latent membrane protein 1 promotes concentration in multivesicular bodies of fibroblast growth factor 2 and its release through exosomes. Int. J. Cancer 121, 1494–1506. 10.1002/ijc.22844 17546597

[B19] Chatenoud L. Bertuccio P. Bosetti C. Malvezzi M. Levi F. Negri E.. (2014). Trends in mortality from major cancers in the Americas: 1980-2010. Ann. Oncol. 25, 1843–1853. 10.1093/annonc/mdu206 24907637

[B20] Chattopadhyay M. K. Jaganandham M. V. (2015). Vesicles-mediated resistance to antibiotics in bacteria. Front. Microbiol. 6:758 10.3389/fmicb.2015.00758 26257725 PMC4511839

[B21] Chen H. Huang J. Wu F. Y. Liao G. Hutt-Fletcher L. Hayward S. D. (2005). Regulation of expression of the Epstein-Barr virus BamHI-A rightward transcripts. J. Virol. 79, 1724–1733. 10.1128/JVI.79.3.1724-1733.2005 15650197 PMC544122

[B22] Chen H. N. Wang Z. Li X. Zhou Z. G. (2016). *Helicobacter pylori* eradication cannot reduce the risk of gastric cancer in patients with intestinal metaplasia and dysplasia: evidence from a meta-analysis. Gastric Cancer 19, 166–175. 10.1007/s10120-015-0462-7 25609452

[B23] Choi H. Lee H. Kim S. R. Gho Y. S. Lee S. K. (2013). Epstein-Barr virus-encoded microRNA BART15-3p promotes cell apoptosis partially by targeting BRUCE. J. Virol. 87, 8135–8144. 10.1128/JVI.03159-12 23678170 PMC3700184

[B24] Choy E. Y. Siu K. L. Kok K. H. Lung R. W. Tsang C. M. To K. F.. (2008). An Epstein-Barr virus-encoded microRNA targets PUMA to promote host cell survival. J. Exp. Med. 205, 2551–2560. 10.1084/jem.20072581 18838543 PMC2571930

[B25] Cohen J. I. (2000). Epstein-Barr virus infection. N. Engl. J. Med. 343, 481–492. 10.1056/NEJM200008173430707 10944566

[B26] Correa P. (1988). Chronic gastritis: a clinico-pathological classification. Am. J. Gastroenterol. 83, 504–509. 3364410

[B27] Cosmopoulos K. Pegtel M. Hawkins J. Moffett H. Novina C. Middeldorp J.. (2009). Comprehensive profiling of Epstein-Barr virus microRNAs in nasopharyngeal carcinoma. J. Virol. 83, 2357–2367. 10.1128/JVI.02104-08 19091858 PMC2643705

[B28] Dela Pena-Ponce M. G. Jimenez M. T. Hansen L. M. Solnick J. V. Miller L. A. (2017). The *Helicobacter pylori* type IV secretion system promotes IL-8 synthesis in a model of pediatric airway epithelium via p38 MAP kinase. PLoS ONE 12:e0183324. 10.1371/journal.pone.0183324 28813514 PMC5557493

[B29] Derrien T. Johnson R. Bussotti G. Tanzer A. Djebali S. Tilgner H.. (2012). The GENCODE v7 catalog of human long noncoding RNAs: analysis of their gene structure, evolution, and expression. Genome Res. 22, 1775–1789. 10.1101/gr.132159.111 22955988 PMC3431493

[B30] Dölken L. Malterer G. Erhard F. Kothe S. Friedel C. C. Suffert G.. (2010). Systematic analysis of viral and cellular microRNA targets in cells latently infected with human gamma-herpesviruses by RISC immunoprecipitation assay. Cell Host Microbe 7, 324–334. 10.1016/j.chom.2010.03.008 20413099

[B31] Du Z. Fei T. Verhaak R. G. Su Z. Zhang Y. Brown M.. (2013). Integrative genomic analyses reveal clinically relevant long noncoding RNAs in human cancer. Nat. Struct. Mol. Biol. 20, 908–913. 10.1038/nsmb.2591 23728290 PMC3702647

[B32] Du Z. M. Hu L. F. Wang H. Y. Yan L. X. Zeng Y. X. Shao J. Y.. (2011). Upregulation of MiR-155 in nasopharyngeal carcinoma is partly driven by LMP1 and LMP2A and downregulates a negative prognostic marker JMJD1A. PLoS ONE 6:e19137. 10.1371/journal.pone.0019137 21541331 PMC3082546

[B33] Dukers D. F. Meij P. Vervoort M. B. Vos W. Scheper R. J. Meijer C. J.. (2000). Direct immunosuppressive effects of EBV-encoded latent membrane protein 1. J. Immunol. 165, 663–670. 10.4049/jimmunol.165.2.663 10878338

[B34] Fassi Fehri L. Koch M. Belogolova E. Khalil H. Bolz C. Kalali B.. (2010). *Helicobacter pylori* induces miR-155 in T cells in a cAMP-Foxp3-dependent manner. PLoS ONE 5:e9500. 10.1371/journal.pone.0009500 20209161 PMC2830477

[B35] Ferlay J. Soerjomataram I. Dikshit R. Eser S. Mathers C. Rebelo M.. (2015). Cancer incidence and mortality worldwide: sources, methods and major patterns in GLOBOCAN 2012. Int. J. Cancer 136, E359–E386. 10.1002/ijc.29210 25220842

[B36] Ferrasi A. C. Pinheiro N. A. Rabenhorst S. H. Caballero O. L. Rodrigues M. A. de Carvalho F.. (2010). *Helicobacter pylori* and EBV in gastric carcinomas: methylation status and microsatellite instability. World J. Gastroenterol. 16, 312–319. 10.3748/wjg.v16.i3.312 20082476 PMC2807951

[B37] Fiocca R. Necchi V. Sommi P. Ricci V. Telford J. Cover T. L.. (1999). Release of *Helicobacter pylori* vacuolating cytotoxin by both a specific secretion pathway and budding of outer membrane vesicles. Uptake of released toxin and vesicles by gastric epithelium. J. Pathol. 188, 220–226. 10.1002/(SICI)1096-9896(199906)188:2<220::AID-PATH307>3.0.CO;2-C 10398168

[B38] Flanagan J. Middeldorp J. Sculley T. (2003). Localization of the Epstein-Barr virus protein LMP 1 to exosomes. J. Gen. Virol. 84, 1871–1879. 10.1099/vir.0.18944-0 12810882

[B39] Franceschi F. Gasbarrini A. (2007). *Helicobacter pylori* and extragastric diseases. Best Pract. Res. Clin. Gastroenterol. 21, 325–334. 10.1016/j.bpg.2006.10.003 17382280

[B40] Franzini M. Corti A. Fierabracci V. Pompella A. (2014). *Helicobacter*, gamma-glutamyltransferase and cancer: further intriguing connections. World J. Gastroenterol. 20, 18057–18058. 10.3748/wjg.v20.i47.18057 25548508 PMC4273160

[B41] Geddert H. zur Hausen A. Gabbert H. E. Sarbia M. (2011). EBV-infection in cardiac and non-cardiac gastric adenocarcinomas is associated with promoter methylation of p16, p14 and APC, but not hMLH1. Cell. Oncol. 34, 209–214. 10.1007/s13402-011-0028-6 PMC13014566 21538028

[B42] Grundhoff A. Sullivan C. S. Ganem D. (2006). A combined computational and microarray-based approach identifies novel microRNAs encoded by human gamma-herpesviruses. RNA 12, 733–750. 10.1261/rna.2326106 16540699 PMC1440911

[B43] Gulley M. L. (2015). Genomic assays for Epstein-Barr virus-positive gastric adenocarcinoma. Exp. Mol. Med. 47:e134. 10.1038/emm.2014.93 25613731 PMC4314585

[B44] Gutiérrez J. Droppelmann C. A. Salsoso R. Westermeier F. Toledo F. Salomon C.. (2016). A hypothesis for the role of RECK in angiogenesis. Curr. Vasc. Pharmacol. 14, 106–115. 10.2174/1570161113666151014130746 26463982

[B45] Hamilton S. Aaltonen L. (2000). Pathology and Genetics of Tumours of the Digestive System. Lyon: IARC Press.

[B46] Haneklaus M. Gerlic M. Kurowska-Stolarska M. Rainey A. A. Pich D. McInnes I. B.. (2012). Cutting edge: miR-223 and EBV miR-BART15 regulate the NLRP3 inflammasome and IL-1beta production. J. Immunol. 189, 3795–3799. 10.4049/jimmunol.1200312 22984081

[B47] Hatakeyama M. (2014). *Helicobacter pylori* CagA and gastric cancer: a paradigm for hit-and-run carcinogenesis. Cell Host Microbe 15, 306–316. 10.1016/j.chom.2014.02.008 24629337

[B48] Hayashi Y. Tsujii M. Wang J. Kondo J. Akasaka T. Jin Y.. (2013). CagA mediates epigenetic regulation to attenuate let-7 expression in *Helicobacter pylori*-related carcinogenesis. Gut 62, 1536–1546. 10.1136/gutjnl-2011-301625 22936674

[B49] He B. Li W. Wu Y. Wei F. Gong Z. Bo H.. (2016). Epstein-Barr virus-encoded miR-BART6-3p inhibits cancer cell metastasis and invasion by targeting long non-coding RNA LOC553103. Cell Death Dis. 7:e2353. 10.1038/cddis.2016.253 27584792 PMC5059857

[B50] He D. Zhang Y. W. Zhang N. N. Zhou L. Chen J. N. Jiang Y.. (2015). Aberrant gene promoter methylation of p16, FHIT, CRBP1, WWOX, and DLC-1 in Epstein-Barr virus-associated gastric carcinomas. Med. Oncol. 32:92. 10.1007/s12032-015-0525-y 25720522

[B51] Helicobacter and Cancer Collaborative Group (2001). Gastric cancer and *Helicobacter pylori*: a combined analysis of 12 case control studies nested within prospective cohorts. Gut 49, 347–353. 10.1136/gut.49.3.347 11511555 PMC1728434

[B52] Hirose T. Mishima Y. Tomari Y. (2014). Elements and machinery of non-coding RNAs: toward their taxonomy. EMBO Rep. 15, 489–507. 10.1002/embr.201338390 24731943 PMC4210095

[B53] Hock B. D. McKenzie J. L. Keenan J. I. (2017). *Helicobacter pylori* outer membrane vesicles inhibit human T cell responses via induction of monocyte COX-2 expression. Pathog. Dis. 75. 10.1093/femspd/ftx034 28430970

[B54] Hurwitz S. N. Cheerathodi M. R. Nkosi D. York S. B. Meckes D. G. Jr. (2018). Tetraspanin CD63 bridges autophagic and endosomal processes to regulate exosomal secretion and intracellular signaling of Epstein-Barr virus LMP1. J. Virol. 92. 10.1128/JVI.01969-17 29212935 PMC5809724

[B55] Hurwitz S. N. Nkosi D. Conlon M. M. York S. B. Liu X. Tremblay D. C.. (2017). CD63 regulates epstein-barr virus LMP1 exosomal packaging, enhancement of vesicle production, and noncanonical NF-κB signaling. J. Virol. 91. 10.1128/JVI.02251-16 27974566 PMC5309960

[B56] Iizasa H. Wulff B. E. Alla N. R. Maragkakis M. Megraw M. Hatzigeorgiou A.. (2010). Editing of Epstein-Barr virus-encoded BART6 microRNAs controls their dicer targeting and consequently affects viral latency. J. Biol. Chem. 285, 33358–33370. 10.1074/jbc.M110.138362 20716523 PMC2963350

[B57] Isomoto H. Matsushima K. Inoue N. Hayashi T. Nakayama T. Kunizaki M.. (2012). Interweaving microRNAs and proinflammatory cytokines in gastric mucosa with reference to H. pylori infection. J. Clin. Immunol. 32, 290–299. 10.1007/s10875-011-9626-3 22161133

[B58] Iwakiri D. Eizuru Y. Tokunaga M. Takada K. (2003). Autocrine growth of Epstein-Barr virus-positive gastric carcinoma cells mediated by an Epstein-Barr virus-encoded small RNA. Cancer Res. 63, 7062–7067. Available online at: http://cancerres.aacrjournals.org/content/63/21/7062.long 14612496

[B59] Iwakiri D. Sheen T. S. Chen J. Y. Huang D. P. Takada K. (2005). Epstein-Barr virus-encoded small RNA induces insulin-like growth factor 1 and supports growth of nasopharyngeal carcinoma-derived cell lines. Oncogene 24, 1767–1773. 10.1038/sj.onc.1208357 15608666

[B60] Jan A. T. (2017). Outer Membrane Vesicles (OMVs) of Gram-negative bacteria: a perspective update. Front. Microbiol. 8:1053. 10.3389/fmicb.2017.01053 28649237 PMC5465292

[B61] Jiang J. Lee E. J. Schmittgen T. D. (2006). Increased expression of microRNA-155 in Epstein-Barr virus transformed lymphoblastoid cell lines. Genes Chromosomes Cancer 45, 103–106. 10.1002/gcc.20264 16175574

[B62] Jones K. R. Whitmire J. M. Merrell D. S. (2010). A tale of two toxins: *Helicobacter Pylori* CagA and VacA modulate host pathways that impact disease. Front. Microbiol. 1:115. 10.3389/fmicb.2010.00115 21687723 PMC3109773

[B63] Jung W. C. Jang Y. J. Kim J. H. Park S. S. Park S. H. Kim S. J.. (2012). Expression of intercellular adhesion molecule-1 and e-selectin in gastric cancer and their clinical significance. J. Gastric Cancer 12, 140–148. 10.5230/jgc.2012.12.3.140 23094225 PMC3473220

[B64] Jung Y. J. Choi H. Kim H. Lee S. K. (2014). MicroRNA miR-BART20-5p stabilizes Epstein-Barr virus latency by directly targeting BZLF1 and BRLF1. J. Virol. 88, 9027–9037. 10.1128/JVI.00721-14 24899173 PMC4136301

[B65] Kanda T. Miyata M. Kano M. Kondo S. Yoshizaki T. Iizasa H. (2015). Clustered microRNAs of the Epstein-Barr virus cooperatively downregulate an epithelial cell-specific metastasis suppressor. J. Virol. 89, 2684–2697. 10.1128/JVI.03189-14 25520514 PMC4325718

[B66] Kang B. W. Choi Y. Kwon O. K. Lee S. S. Chung H. Y. Yu W.. (2017). High level of viral microRNA-BART20-5p expression is associated with worse survival of patients with Epstein-Barr virus-associated gastric cancer. Oncotarget 8, 14988–14994. 10.18632/oncotarget.14744 28122341 PMC5362460

[B67] Kang D. Skalsky R. L. Cullen B. R. (2015). EBV BART MicroRNAs target multiple pro-apoptotic cellular genes to promote epithelial cell survival. PLoS Pathog. 11:e1004979. 10.1371/journal.ppat.1004979 26070070 PMC4466530

[B68] Kim D. N. Chae H. S. Oh S. T. Kang J. H. Park C. H. Park W. S.. (2007). Expression of viral microRNAs in Epstein-Barr virus-associated gastric carcinoma. J. Virol. 81, 1033–1036. 10.1128/JVI.02271-06 17079300 PMC1797424

[B69] Kim D. N. Lee S. K. (2012). Biogenesis of Epstein-Barr virus microRNAs. Mol. Cell. Biochem. 365, 203–210. 10.1007/s11010-012-1261-7 22350759

[B70] Kim D. N. Seo M. K. Choi H. Kim S. Y. Shin H. J. Yoon A. R.. (2013). Characterization of naturally Epstein-Barr virus-infected gastric carcinoma cell line YCCEL1. J. Gen. Virol. 94, 497–506. 10.1099/vir.0.045237-0 23175241

[B71] Kim H. Choi H. Lee S. K. (2015). Epstein-Barr virus miR-BART20-5p regulates cell proliferation and apoptosis by targeting BAD. Cancer Lett. 356, 733–742. 10.1016/j.canlet.2014.10.023 25449437

[B72] Kim S. M. Hur D. Y. Hong S. W. Kim J. H. (2017). EBV-encoded EBNA1 regulates cell viability by modulating miR34a-NOX2-ROS signaling in gastric cancer cells. Biochem. Biophys. Res. Commun. 494, 550–555. 10.1016/j.bbrc.2017.10.095 29061308

[B73] Ko S. H. Jeon J. I. Kim Y. J. Yoon H. J. Kim H. Kim N.. (2015). *Helicobacter pylori* outer membrane vesicle proteins induce human eosinophil degranulation via a beta2 Integrin CD11/CD18- and ICAM-1-dependent mechanism. Mediators Inflamm. 2015:301716. 10.1155/2015/301716 25821353 PMC4364020

[B74] Koch M. Mollenkopf H. J. Klemm U. Meyer T. F. (2012). Induction of microRNA-155 is TLR- and type IV secretion system-dependent in macrophages and inhibits DNA-damage induced apoptosis. Proc. Natl. Acad. Sci. U.S.A. 109, E1153–E1162. 10.1073/pnas.1116125109 22509021 PMC3358876

[B75] Kowal J. Tkach M. Théry C. (2014). Biogenesis and secretion of exosomes. Curr. Opin. Cell Biol. 29, 116–125. 10.1016/j.ceb.2014.05.004 24959705

[B76] Lauren P. (1965). The two histological main types of gastric carcinoma: diffuse and so-called intestinal-type carcinoma. an attempt at a histo-clinical classification. Acta Pathol. Microbiol. Scand. 64, 31–49. 10.1111/apm.1965.64.1.31 14320675

[B77] Lei T. Yuen K. S. Xu R. Tsao S. W. Chen H. Li M.. (2013). Targeting of DICE1 tumor suppressor by Epstein-Barr virus-encoded miR-BART3^*^ microRNA in nasopharyngeal carcinoma. Int. J. Cancer 133, 79–87. 10.1002/ijc.28007 23280823

[B78] Lerner M. R. Andrews N. C. Miller G. Steitz J. A. (1981). Two small RNAs encoded by Epstein-Barr virus and complexed with protein are precipitated by antibodies from patients with systemic lupus erythematosus. Proc. Natl. Acad. Sci. U.S.A. 78, 805–809. 10.1073/pnas.78.2.805 6262773 PMC319891

[B79] Li T. Mo X. Fu L. Xiao B. Guo J. (2016). Molecular mechanisms of long noncoding RNAs on gastric cancer. Oncotarget 7, 8601–8612. 10.18632/oncotarget.6926 26788991 PMC4890990

[B80] Liao Q. Zeng Z. Guo X. Li X. Wei F. Zhang W.. (2014). LPLUNC1 suppresses IL-6-induced nasopharyngeal carcinoma cell proliferation via inhibiting the Stat3 activation. Oncogene 33, 2098–2109. 10.1038/onc.2013.161 23708661

[B81] Liu J. Y. Peng C. W. Yang G. F. Hu W. Q. Yang X. J. Huang C. Q.. (2017). Distribution pattern of tumor associated macrophages predicts the prognosis of gastric cancer. Oncotarget 8, 92757–92769. 10.18632/oncotarget.21575 29190953 PMC5696219

[B82] Liu X. Cohen J. I. (2015). Epstein-Barr Virus (EBV) Tegument protein BGLF2 promotes EBV reactivation through activation of the p38 mitogen-activated protein kinase. J. Virol. 90, 1129–1138. 10.1128/JVI.01410-15 26559845 PMC4702675

[B83] Lo A. K. To K. F. Lo K. W. Lung R. W. Hui J. W. Liao G.. (2007). Modulation of LMP1 protein expression by EBV-encoded microRNAs. Proc. Natl. Acad. Sci. U.S.A. 104, 16164–16169. 10.1073/pnas.0702896104 17911266 PMC2042179

[B84] Lung R. W. Tong J. H. Sung Y. M. Leung P. S. Ng D. C. Chau S. L.. (2009). Modulation of LMP2A expression by a newly identified Epstein-Barr virus-encoded microRNA miR-BART22. Neoplasia 11, 1174–1184. 10.1593/neo.09888 19881953 PMC2767219

[B85] Mansouri S. Pan Q. Blencowe B. J. Claycomb J. M. Frappier L. (2014). Epstein-Barr virus EBNA1 protein regulates viral latency through effects on let-7 microRNA and dicer. J. Virol. 88, 11166–11177. 10.1128/JVI.01785-14 25031339 PMC4178782

[B86] Marquitz A. R. Mathur A. Chugh P. E. Dittmer D. P. Raab-Traub N. (2014). Expression profile of microRNAs in Epstein-Barr virus-infected AGS gastric carcinoma cells. J. Virol. 88, 1389–1393. 10.1128/JVI.02662-13 24227849 PMC3911632

[B87] Marquitz A. R. Mathur A. Nam C. S. Raab-Traub N. (2011). The Epstein-Barr virus BART microRNAs target the pro-apoptotic protein Bim. Virology 412, 392–400. 10.1016/j.virol.2011.01.028 21333317 PMC3340891

[B88] Marquitz A. R. Mathur A. Shair K. H. Raab-Traub N. (2012). Infection of Epstein-Barr virus in a gastric carcinoma cell line induces anchorage independence and global changes in gene expression. Proc. Natl. Acad. Sci. U.S.A. 109, 9593–9598. 10.1073/pnas.1202910109 22647604 PMC3386136

[B89] Matsushima K. Isomoto H. Inoue N. Nakayama T. Hayashi T. Nakayama M.. (2011). MicroRNA signatures in *Helicobacter pylori*-infected gastric mucosa. Int. J. Cancer 128, 361–370. 10.1002/ijc.25348 20333682

[B90] Meckes D. G. Jr. (2015). Exosomal communication goes viral. J. Virol. 89, 5200–5203. 10.1128/JVI.02470-14 25740980 PMC4442506

[B91] Meckes D. G. Jr. Gunawardena H. P. Dekroon R. M. Heaton P. R. Edwards R. H. Ozgur S.. (2013). Modulation of B-cell exosome proteins by gamma herpesvirus infection. Proc. Natl. Acad. Sci. U.S.A. 110, E2925–E2933. 10.1073/pnas.1303906110 23818640 PMC3732930

[B92] Meckes D. G. Jr. Raab-Traub N. (2011). Microvesicles and viral infection. J. Virol. 85, 12844–12854. 10.1128/JVI.05853-11 21976651 PMC3233125

[B93] Meckes D. G. Jr. Shair K. H. Marquitz A. R. Kung C. P. Edwards R. H. Raab-Traub N. (2010). Human tumor virus utilizes exosomes for intercellular communication. Proc. Natl. Acad. Sci. U.S.A. 107, 20370–20375. 10.1073/pnas.1014194107 21059916 PMC2996715

[B94] Mohr C. F. Kalmer M. Gross C. Mann M. C. Sterz K. R. Kieser A.. (2014). The tumor marker Fascin is induced by the Epstein-Barr virus-encoded oncoprotein LMP1 via NF-kappaB in lymphocytes and contributes to their invasive migration. Cell Commun. Signal. 12:46. 10.1186/s12964-014-0046-x 25105941 PMC4222691

[B95] Morson B. C. (1955). Intestinal metaplasia of the gastric mucosa. Br. J. Cancer 9, 365–376. 10.1038/bjc.1955.35 13269634 PMC2073705

[B96] Moss S. F. (2017). The clinical evidence linking *Helicobacter pylori* to gastric cancer. Cell Mol. Gastroenterol. Hepatol. 3, 183–191. 10.1016/j.jcmgh.2016.12.001 28275685 PMC5331857

[B97] Murray-Stewart T. Sierra J. C. Piazuelo M. B. Mera R. M. Chaturvedi R. Bravo L. E.. (2016). Epigenetic silencing of miR-124 prevents spermine oxidase regulation: implications for *Helicobacter pylori*-induced gastric cancer. Oncogene 35, 5480–5488. 10.1038/onc.2016.91 27041578 PMC5050049

[B98] Nachmani D. Stern-Ginossar N. Sarid R. Mandelboim O. (2009). Diverse herpesvirus microRNAs target the stress-induced immune ligand MICB to escape recognition by natural killer cells. Cell Host Microbe. 5, 376–385. 10.1016/j.chom.2009.03.003 19380116

[B99] Nolte-'t Hoen E. Cremer T. Gallo R. C. Margolis L. B. (2016). Extracellular vesicles and viruses: are they close relatives? Proc. Natl. Acad. Sci. U.S.A. 113, 9155–9161. 10.1073/pnas.1605146113 27432966 PMC4995926

[B100] Olofsson A. Vallström A. Petzold K. Tegtmeyer N. Schleucher J. Carlsson S.. (2010). Biochemical and functional characterization of *Helicobacter pylori* vesicles. Mol. Microbiol. 77, 1539–1555. 10.1111/j.1365-2958.2010.07307.x 20659286 PMC3068288

[B101] Parker H. Keenan J. I. (2012). Composition and function of *Helicobacter pylori* outer membrane vesicles. Microbes Infect. 14, 9–16. 10.1016/j.micinf.2011.08.007 21911076

[B102] Pegtel D. M. Cosmopoulos K. Thorley-Lawson D. A. van Eijndhoven M. A. Hopmans E. S. Lindenberg J. L.. (2010). Functional delivery of viral miRNAs via exosomes. Proc. Natl. Acad. Sci. U.S.A. 107, 6328–6333. 10.1073/pnas.0914843107 20304794 PMC2851954

[B103] Pfeffer S. Sewer A. Lagos-Quintana M. Sheridan R. Sander C. Grässer F. A.. (2005). Identification of microRNAs of the herpesvirus family. Nat. Methods 2, 269–276. 10.1038/nmeth746 15782219

[B104] Pfeffer S. Zavolan M. Grässer F. A. Chien M. Russo J. J. Ju J.. (2004). Identification of virus-encoded microRNAs. Science 304, 734–736. 10.1126/science.1096781 15118162

[B105] Price A. M. Luftig M. A. (2015). To be or not IIb: a multi-step process for Epstein-Barr virus latency establishment and consequences for B cell tumorigenesis. PLoS Pathog. 11:e1004656. 10.1371/journal.ppat.1004656 25790223 PMC4366242

[B106] Qiu J. Cosmopoulos K. Pegtel M. Hopmans E. Murray P. Middeldorp J.. (2011). A novel persistence associated EBV miRNA expression profile is disrupted in neoplasia. PLoS Pathog. 7:e1002193. 10.1371/journal.ppat.1002193 21901094 PMC3161978

[B107] Raab-Traub N. Dittmer D. P. (2017). Viral effects on the content and function of extracellular vesicles. Nat. Rev. Microbiol. 15, 559–572. 10.1038/nrmicro.2017.60 28649136 PMC5555775

[B108] Ribeiro J. Oliveira C. Malta M. Sousa H. (2017). Epstein-Barr virus gene expression and latency pattern in gastric carcinomas: a systematic review. Future Oncol. 13, 567–579. 10.2217/fon-2016-0475 28118740

[B109] Ricci V. Chiozzi V. Necchi V. Oldani A. Romano M. Solcia E.. (2005). Free-soluble and outer membrane vesicle-associated VacA from *Helicobacter pylori*: two forms of release, a different activity. Biochem. Biophys. Res. Commun. 337, 173–178. 10.1016/j.bbrc.2005.09.035 16182250

[B110] Ricci V. Giannouli M. Romano M. Zarrilli R. (2014). *Helicobacter pylori* gamma-glutamyl transpeptidase and its pathogenic role. World J. Gastroenterol. 20, 630–638. 10.3748/wjg.v20.i3.630 24574736 PMC3921472

[B111] Rider M. A. Cheerathodi M. R. Hurwitz S. N. Nkosi D. Howell L. A. Tremblay D. C.. (2018). The interactome of EBV LMP1 evaluated by proximity-based BioID approach. Virology 516, 55–70. 10.1016/j.virol.2017.12.033 29329079 PMC5826876

[B112] Riley K. J. Rabinowitz G. S. Yario T. A. Luna J. M. Darnell R. B. Steitz J. A. (2012). EBV and human microRNAs co-target oncogenic and apoptotic viral and human genes during latency. EMBO J. 31, 2207–2221. 10.1038/emboj.2012.63 22473208 PMC3343464

[B113] Risch H. A. Lu L. Kidd M. S. Wang J. Zhang W. Ni Q.. (2014). *Helicobacter pylori* seropositivities and risk of pancreatic carcinoma. Cancer Epidemiol. Biomarkers Prev. 23, 172–178. 10.1158/1055-9965.EPI-13-0447 24234587 PMC3947155

[B114] Rosa M. D. Gottlieb E. Lerner M. R. Steitz J. A. (1981). Striking similarities are exhibited by two small Epstein-Barr virus-encoded ribonucleic acids and the adenovirus-associated ribonucleic acids VAI and VAII. Mol. Cell. Biol. 1, 785–796. 10.1128/MCB.1.9.785 9279391 PMC369362

[B115] Rymo L. (1979). Identification of transcribed regions of Epstein-Barr virus DNA in Burkitt lymphoma-derived cells. J. Virol. 32, 8–18. 232190 10.1128/jvi.32.1.8-18.1979 PMC353521

[B116] Saito M. Nishikawa J. Okada T. Morishige A. Sakai K. Nakamura M.. (2013). Role of DNA methylation in the development of Epstein-Barr virus-associated gastric carcinoma. J. Med. Virol. 85, 121–127. 10.1002/jmv.23405 23073987

[B117] Saju P. Murata-Kamiya N. Hayashi T. Senda Y. Nagase L. Noda S.. (2016). Host SHP1 phosphatase antagonizes *Helicobacter pylori* CagA and can be downregulated by Epstein-Barr virus. Nat. Microbiol. 1:16026. 10.1038/nmicrobiol.2016.26 27572445

[B118] Samanta M. Iwakiri D. Kanda T. Imaizumi T. Takada K. (2006). EB virus-encoded RNAs are recognized by RIG-I and activate signaling to induce type I IFN. EMBO J. 25, 4207–4214. 10.1038/sj.emboj.7601314 16946700 PMC1570431

[B119] Sana J. Faltejskova P. Svoboda M. Slaby O. (2012). Novel classes of non-coding RNAs and cancer. J. Transl. Med. 10:103. 10.1186/1479-5876-10-103 22613733 PMC3434024

[B120] Sandoval-Bórquez A. Saavedra K. Carrasco-Avino G. Garcia-Bloj B. Fry J. Wichmann I.. (2015). Noncoding genomics in Gastric Cancer and the gastric precancerous cascade: pathogenesis and biomarkers. Dis. Markers 2015:503762. 10.1155/2015/503762 26379360 PMC4563069

[B121] Sato Y. Ochiai S. Murata T. Kanda T. Goshima F. Kimura H. (2017). Elimination of LMP1-expressing cells from a monolayer of gastric cancer AGS cells. Oncotarget 8, 39345–39355. 10.18632/oncotarget.16996 28454117 PMC5503617

[B122] Schmitt A. M. Chang H. Y. (2016). Long Noncoding RNAs in Cancer pathways. Cancer Cell. 29, 452–463. 10.1016/j.ccell.2016.03.010 27070700 PMC4831138

[B123] Schneider B. G. Peng D. F. Camargo M. C. Piazuelo M. B. Sicinschi L. A. Mera R.. (2010). Promoter DNA hypermethylation in gastric biopsies from subjects at high and low risk for gastric cancer. Int. J. Cancer 127, 2588–2597. 10.1002/ijc.25274 20178103 PMC2916942

[B124] Seto E. Moosmann A. Grömminger S. Walz N. Grundhoff A. Hammerschmidt W. (2010). Micro RNAs of Epstein-Barr virus promote cell cycle progression and prevent apoptosis of primary human B cells. PLoS Pathog. 6:e1001063. 10.1371/journal.ppat.1001063 20808852 PMC2924374

[B125] Shibata D. Weiss L. M. (1992). Epstein-Barr virus-associated gastric adenocarcinoma. Am. J. Pathol. 140, 769–774. 1314023 PMC1886378

[B126] Shimoda A. Ueda K. Nishiumi S. Murata-Kamiya N. Mukai S. A. Sawada S.. (2016). Exosomes as nanocarriers for systemic delivery of the *Helicobacter pylori* virulence factor CagA. Sci. Rep. 6:18346. 10.1038/srep18346 26739388 PMC4703974

[B127] Shinozaki A. Sakatani T. Ushiku T. Hino R. Isogai M. Ishikawa S.. (2010). Downregulation of microRNA-200 in EBV-associated gastric carcinoma. Cancer Res. 70, 4719–4727. 10.1158/0008-5472.CAN-09-4620 20484038

[B128] Shinozaki-Ushiku A. Kunita A. Isogai M. Hibiya T. Ushiku T. Takada K.. (2015). Profiling of virus-encoded microRNAs in Epstein-Barr virus-associated gastric carcinoma and their roles in gastric carcinogenesis. J. Virol. 89, 5581–5591. 10.1128/JVI.03639-14 25740983 PMC4442544

[B129] Song Y. Li X. Zeng Z. Li Q. Gong Z. Liao Q.. (2016). Epstein-Barr virus encoded miR-BART11 promotes inflammation-induced carcinogenesis by targeting FOXP1. Oncotarget 7, 36783–36799. 10.18632/oncotarget.9170 27167345 PMC5095039

[B130] Strofilas A. Lagoudianakis E. E. Seretis C. Pappas A. Koronakis N. Keramidaris D.. (2012). Association of helicobacter pylori infection and colon cancer. J. Clin. Med. Res. 4, 172–176. 10.4021/jocmr880w 22719803 PMC3376875

[B131] Sudo M. Chong J. M. Sakuma K. Ushiku T. Uozaki H. Nagai H.. (2004). Promoter hypermethylation of E-cadherin and its abnormal expression in Epstein-Barr virus-associated gastric carcinoma. Int. J. Cancer 109, 194–199. 10.1002/ijc.11701 14750169

[B132] Sun J. Xu K. Wu C. Wang Y. Hu Y. Zhu Y.. (2007). PD-L1 expression analysis in gastric carcinoma tissue and blocking of tumor-associated PD-L1 signaling by two functional monoclonal antibodies. Tissue Antigens 69, 19–27. 10.1111/j.1399-0039.2006.00701.x 17212704

[B133] Tagawa T. Albanese M. Bouvet M. Moosmann A. Mautner J. Heissmeyer V.. (2016). Epstein-Barr viral miRNAs inhibit antiviral CD4+ T cell responses targeting IL-12 and peptide processing. J. Exp. Med. 213, 2065–2208. 10.1084/jem.20160248 27621419 PMC5030804

[B134] Teng G. G. Wang W. H. Dai Y. Wang S. J. Chu Y. X. Li J. (2013). Let-7b is involved in the inflammation and immune responses associated with *Helicobacter pylori* infection by targeting Toll-like receptor 4. PLoS ONE 8:e56709. 10.1371/journal.pone.0056709 23437218 PMC3577724

[B135] The Cancer Genome Atlas Research Network T. (2014). Comprehensive molecular characterization of gastric adenocarcinoma. Nature 513, 202–209. 10.1038/nature13480 25079317 PMC4170219

[B136] Torre L. A. Bray F. Siegel R. L. Ferlay J. Lortet-Tieulent J. Jemal A. (2015). Global cancer statistics, (2012). CA Cancer J. Clin. 65, 87–108. 10.3322/caac.21262 25651787

[B137] Treece A. L. Duncan D. L. Tang W. Elmore S. Morgan D. R. Dominguez R. L.. (2016). Gastric adenocarcinoma microRNA profiles in fixed tissue and in plasma reveal cancer-associated and Epstein-Barr virus-related expression patterns. Lab. Invest. 96, 661–671. 10.1038/labinvest.2016.33 26950485 PMC5767475

[B138] Tsai C. Y. Liu Y. Y. Liu K. H. Hsu J. T. Chen T. C. Chiu C. T.. (2017). Comprehensive profiling of virus microRNAs of Epstein-Barr virus-associated gastric carcinoma: highlighting the interactions of ebv-Bart9 and host tumor cells. J. Gastroenterol. Hepatol. 32, 82–91. 10.1111/jgh.13432 27144885

[B139] Vallhov H. Gutzeit C. Johansson S. M. Nagy N. Paul M. Li Q.. (2011). Exosomes containing glycoprotein 350 released by EBV-transformed B cells selectively target B cells through CD21 and block EBV infection *in vitro*. J. Immunol. 186, 73–82. 10.4049/jimmunol.1001145 21106852

[B140] van Dongen H. M. Masoumi N. Witwer K. W. Pegtel D. M. (2016). Extracellular vesicles exploit viral entry routes for cargo delivery. Microbiol. Mol. Biol. Rev. 80, 369–386. 10.1128/MMBR.00063-15 26935137 PMC4867369

[B141] Vereide D. T. Seto E. Chiu Y. F. Hayes M. Tagawa T. Grundhoff A.. (2013). Epstein-Barr virus maintains lymphomas via its miRNAs. Oncogene 33, 1258–1264. 10.1038/onc.2013.71 23503461 PMC3690170

[B142] Wang J. Wu J. Cheng Y. Jiang Y. Li G. (2016). Over-expression of microRNA-223 inhibited the proinflammatory responses in *Helicobacter pylori*-infection macrophages by down-regulating IRAK-1. Am. J. Transl. Res. 8, 615–622. 27158353 PMC4846910

[B143] Weiss L. M. Chen Y. Y. (2013). EBER in situ hybridization for Epstein-Barr virus. Methods Mol. Biol. 999, 223–230. 10.1007/978-1-62703-357-2_16 23666702

[B144] Winter J. Letley D. Rhead J. Atherton J. Robinson K. (2014). *Helicobacter pylori* membrane vesicles stimulate innate pro- and anti-inflammatory responses and induce apoptosis in Jurkat T cells. Infect. Immun. 82, 1372–1381. 10.1128/IAI.01443-13 24421041 PMC3993389

[B145] Wong A. M. Kong K. L. Tsang J. W. Kwong D. L. Guan X. Y. (2012). Profiling of Epstein-Barr virus-encoded microRNAs in nasopharyngeal carcinoma reveals potential biomarkers and oncomirs. Cancer 118, 698–711. 10.1002/cncr.26309 21720996

[B146] Wroblewski L. E. Peek R. M. Jr. (2013). *Helicobacter pylori* in gastric carcinogenesis: mechanisms. Gastroenterol. Clin. North Am. 42, 285–298. 10.1016/j.gtc.2013.01.006 23639641 PMC3648881

[B147] Xiao B. Liu Z. Li B. S. Tang B. Li W. Guo G.. (2009). Induction of microRNA-155 during *Helicobacter pylori* infection and its negative regulatory role in the inflammatory response. J. Infect. Dis. 200, 916–925. 10.1086/605443 19650740

[B148] Yang F. Xu Y. Liu C. Ma C. Zou S. Xu X.. (2018). NF-kappaB/miR-223-3p/ARID1A axis is involved in *Helicobacter pylori* CagA-induced gastric carcinogenesis and progression. Cell Death Dis. 9:12. 10.1038/s41419-017-0020-9 29317648 PMC5849037

[B149] Yang L. Long Y. Li C. Cao L. Gan H. Huang K.. (2015). Genome-wide analysis of long noncoding RNA profile in human gastric epithelial cell response to *Helicobacter pylori*. Jpn. J. Infect. Dis. 68, 63–66. 10.7883/yoken.JJID.2014.149 25420666

[B150] Yau T. O. Tang C. M. Yu J. (2014). Epigenetic dysregulation in Epstein-Barr virus-associated gastric carcinoma: disease and treatments. World J. Gastroenterol. 20, 6448–6456. 10.3748/wjg.v20.i21.6448 24914366 PMC4047330

[B151] Yong X. Tang B. Li B. S. Xie R. Hu C. J. Luo G.. (2015). *Helicobacter pylori* virulence factor CagA promotes tumorigenesis of gastric cancer via multiple signaling pathways. Cell Commun. Signal. 13:30. 10.1186/s12964-015-0111-0 26160167 PMC4702319

[B152] Yoshida N. Kimura T. (2017). Pathogen-associated regulatory non-coding RNAs and oncogenesis. Front. Biosci. 22, 1599–1621. 10.2741/4560 28410134

[B153] Young L. S. Yap L. F. Murray P. G. (2016). Epstein-Barr virus: more than 50 years old and still providing surprises. Nat. Rev. Cancer 16, 789–802. 10.1038/nrc.2016.92 27687982

[B154] Yu X. W. Xu Q. Xu Y. Gong Y. H. Yuan Y. (2014). Expression of the E-cadherin/β-catenin/tcf-4 pathway in gastric diseases with relation to *Helicobacter pylori* infection: clinical and pathological implications. Asian Pac. J. Cancer Prev. 15, 215–220. 10.7314/APJCP.2014.15.1.215 24528029

[B155] Yu X. W. Xu Y. Gong Y. H. Qian X. Yuan Y. (2011). *Helicobacter pylori* induces malignant transformation of gastric epithelial cells *in vitro*. APMIS 119, 187–197. 10.1111/j.1600-0463.2010.02709.x 21284736

[B156] Zhang B. G. Hu L. Zang M. D. Wang H. X. Zhao W. Li J. F.. (2016). *Helicobacter pylori* CagA induces tumor suppressor gene hypermethylation by upregulating DNMT1 via AKT-NFkappaB pathway in gastric cancer development. Oncotarget 7, 9788–9980. 10.18632/oncotarget.7125 26848521 PMC4891084

[B157] Zhang G. Ducatelle R. Pasmans F. D'Herde K. Huang L. Smet A.. (2013). Effects of Helicobacter suis gamma-glutamyl transpeptidase on lymphocytes: modulation by glutamine and glutathione supplementation and outer membrane vesicles as a putative delivery route of the enzyme. PLoS ONE 8:e77966. 10.1371/journal.pone.0077966 24147103 PMC3797756

[B158] Zhang J. Yuan Y. Wei Z. Ren J. Hou X. Yang D.. (2018). Crosstalk between prognostic long noncoding RNAs and messenger RNAs as transcriptional hallmarks in gastric cancer. Epigenomics [Epub ahead of print]. 10.2217/epi-2017-0136 29402138

[B159] Zhang Z. Li Z. Gao C. Chen P. Chen J. Liu W.. (2008). miR-21 plays a pivotal role in gastric cancer pathogenesis and progression. Lab. Invest. 88, 1358–1366. 10.1038/labinvest.2008.94 18794849

[B160] Zhang Z. Li Z. Li Y. Zang A. (2014). MicroRNA and signaling pathways in gastric cancer. Cancer Gene Ther. 21, 305–316. 10.1038/cgt.2014.37 25060632

[B161] Zhou X. Chen H. Zhu L. Hao B. Zhang W. Hua J.. (2016). *Helicobacter pylori* infection related long noncoding RNA (lncRNA) AF147447 inhibits gastric cancer proliferation and invasion by targeting MUC2 and up-regulating miR-34c. Oncotarget 7, 82770–82782. 10.18632/oncotarget.13165 27835575 PMC5347731

[B162] Zhu H. Wang Q. Yao Y. Fang J. Sun F. Ni Y.. (2015). Microarray analysis of Long non-coding RNA expression profiles in human gastric cells and tissues with *Helicobacter pylori* Infection. BMC Med. Genomics 8:84. 10.1186/s12920-015-0159-0 26690385 PMC4687289

[B163] Zhu J. Y. Pfuhl T. Motsch N. Barth S. Nicholls J. Grasser F.. (2009). Identification of novel Epstein-Barr virus microRNA genes from nasopharyngeal carcinomas. J. Virol. 83, 3333–3341. 10.1128/JVI.01689-08 19144710 PMC2655542

[B164] Zouridis H. Deng N. Ivanova T. Zhu Y. Wong B. Huang D.. (2012). Methylation subtypes and large-scale epigenetic alterations in gastric cancer. Sci. Transl. Med. 4:156ra140. 10.1126/scitranslmed.3004504 23076357

[B165] zur Hausen A. Brink A. A. Craanen M. E. Middeldorp J. M. Meijer C. J. van den Brule A. J. (2000). Unique transcription pattern of Epstein-Barr virus (EBV) in EBV-carrying gastric adenocarcinomas: expression of the transforming BARF1 gene. Cancer Res. 60, 2745–2748. Available online at: http://cancerres.aacrjournals.org/content/60/10/2745.long 10825150

